# Differentiation resistance through altered retinoblastoma protein function in acute lymphoblastic leukemia: in silico modeling of the deregulations in the G1/S restriction point pathway

**DOI:** 10.1186/s12918-016-0264-5

**Published:** 2016-03-01

**Authors:** Eleftherios Ouzounoglou, Dimitra Dionysiou, Georgios S. Stamatakos

**Affiliations:** In Silico Oncology and In Silico Medicine Group, Laboratory of Microwaves and Fiber Optics, Institute of Communication and Computer Systems, School of Electrical and Computer Engineering, National Technical University of Athens, 9 Iroon Polytechniou, Zografou, 15780 Athens, Greece

**Keywords:** Cell cycle, Acute lymphoblastic leukemia, Restriction point, Retinoblastoma protein, In silico modeling, In silico oncology, Computational oncology, In silico medicine, Cancer multiscale modeling, Cancer systems biology

## Abstract

**Background:**

As in many cancer types, the G1/S restriction point (RP) is deregulated in Acute Lymphoblastic Leukemia (ALL). Hyper-phosphorylated retinoblastoma protein (hyper-pRb) is found in high levels in ALL cells. Nevertheless, the ALL lymphocyte proliferation rate for the average patient is surprisingly low compared to its normal counterpart of the same maturation level. Additionally, as stated in literature, ALL cells possibly reside at or beyond the RP which is located in the late-G1 phase. This state may favor their differentiation resistant phenotype. A major phenomenon contributing to this fact is thought to be the observed limited redundancy in the phosphorylation of retinoblastoma protein (pRb) by the various Cyclin Dependent Kinases (Cdks). The latter may result in partial loss of pRb functions despite hyper-phosphorylation.

**Results:**

To test this hypothesis, an *in silico* model aiming at simulating the biochemical regulation of the RP in ALL is introduced. By exploiting experimental findings derived from leukemic cells and following a semi-quantitative calibration procedure, the model has been shown to satisfactorily reproduce such a behavior for the RP pathway. At the same time, the calibrated model has been proved to be in agreement with the observed variation in the ALL cell cycle duration.

**Conclusions:**

The proposed model aims to contribute to a better understanding of the complex phenomena governing the leukemic cell cycle. At the same time it constitutes a significant first step in the creation of a personalized proliferation rate predictor that can be used in the context of multiscale cancer modeling. Such an approach is expected to play an important role in the refinement and the advancement of mechanistic modeling of ALL in the context of the emergent and promising scientific domains of *In Silico* Oncology and more generally *In Silico* Medicine.

**Electronic supplementary material:**

The online version of this article (doi:10.1186/s12918-016-0264-5) contains supplementary material, which is available to authorized users.

## Background

Multiscale cancer modeling is a rapidly growing field that gradually attracts interest from many researchers in computational and life sciences. The central objective and vision of this discipline could be distilled into the creation of models supporting our understanding of the natural phenomenon of cancer. The latter is also paving the way for the newly emerged scientific fields of *In Silico* Oncology and *In Silico* Medicine [1, 2].

Cancer is a multiscale biological phenomenon manifested in the molecular, cellular, tissue, organ or even whole organism levels. Therefore, *In Silico* cancer models should be developed in a way to reflect this diversity of bio-complexity scales. In this context, the development of a proper methodology and technology infrastructure that will allow the effective combination of different cancer related (sub-) models into multiscale hyper-models is the central objective of the European Commission (EC) funded Project “Computational Horizons In Cancer (CHIC)” (FP7-ICT-2011-9, Grant agreement no: 600841). Additionally, the high heterogeneity among different cancer types (or even sub-types) should be incorporated into models. Thus, (sub-) models that refer to the same type of cancer should be created, if not already available. This can be done either from scratch or by modifying already existing models, e.g. by introducing experimental findings for the specific biological phenomenon of interest.

In this setting, a model that is capable of simulating the sub-cellular biochemical dynamics regulating the cell cycle in Acute Lymphoblastic Leukemia (ALL) is proposed. The mid-term purpose of the model development is to be coupled with the ALL Oncosimulator [3–5], developed in the framework of the European Commission (EC) funded project p-medicine (FP7-ICT-2009.5.3 -*270089*) by the *In Silico* Oncology and *In Silico* Medicine Group (ISO&ISM_G), Institute of Communication and Computer Systems (ICCS), National Technical University of Athens (NTUA). The Oncosimulator [1, 6–9] as a modeling concept and system focuses on the simulation of cancer growth and response to treatment in the patient individualized context. Many other versions of the ISO&ISM_G Oncosimulator have been defined and implemented during the last years in the framework of the EC funded projects ACGT (FP6-2005-IST-026996), Contra Cancrum (FP7-ICT-2007-2-223979) and TUMOR (FP7-ICT-2009.5.4-247754) and have dealt with various types of human tumors. In the development and clinical adaptation of the Oncosimulators, clinically available data are used extensively.

One of the most significant input parameters of the ISO&ISM_G ALL Oncosimulator is the cell cycle duration of tumor cells *T*_*c*_ [10–12]. The latter highlights the need for a detailed study of the leukemic cell cycle.

ALL is the most common neoplastic malignancy in children, the acuteness of which results from the resistance of ALL cells to differentiation stimuli [13]. This non-solid hematological cancer is characterized by a huge immunological and genomic heterogeneity of the transformed cells (diverse lineages of malignant cells, either B-cells or T-cells, and specific chromosomal and genetic abnormalities [14, 15]). In the context of the present study we have focused, to the extent possible, on the precursor B Acute Lymphoblastic Leukemia (BCP-ALL) subtype. This choice has been made not only due to the high incidence rate of this subtype [16, 17], but also because of the substantial amount of related knowledge accumulated in literature.

BCP-ALL cells show some remarkable cell cycle characteristics in various levels of bio-complexity. The cancer stem cell hypothesis [18–20] has been recently questioned for the case of ALL [21, 22]. At the same time BCP-ALL subpopulations with very different cell cycle kinetics have been found in bone marrow samples [23]. Specifically, CD19+ cells (expressing the B-cell antigen CD19) are the dominant and most proliferative cells in BCP-ALL samples, constituting more than 90 % of the entire population [23]. It is stressed that in order to formulate the proposed model, the assumption that all the information extracted from literature mainly refers to CD19+ cells has been made.

Focusing on the cellular level, leukemic cells from BCP-ALL patients show a mean (±standard deviation) *T*_*c*_ value of 112.5 (±46.8) hours (*h*) compared to the 65.5 (±3.5) *h* value of their normal counterparts (non-neoplastic precursor B cells) [24]. Concerning the distribution of BCP-ALL cells in the cell cycle phases, it has been shown that the majority of cells reside in the G1 phase (more than 80 %) and only a minor proportion can be found in S (~7–10 %) and G2/M phases [25–27]. Moreover, the percentage of quiescent cells (found in true G0) is really low (~2 %) [28, 29]. Finally, another characteristic of BCP-ALL cells that could explain their almost complete dominance in patients’ bone marrow, despite their low proliferation rate, is the significantly reduced incidence of apoptosis. This route of cell death typically reduces the leukemic cell mass by 4 % per day, while cell birth results in an average of 10–11 % daily expansion [30].

Moving deeper into processes at the molecular level and focusing on the metabolism of cells, the switch to aerobic glycolysis (known as Warburg effect), which is commonly observed in cancers, has also been shown to be manifested in ALL [31]. Focusing on processes directly regulating the cell cycle, a finding of great importance is the almost exclusively hyper-phosphorylated status of retinoblastoma protein (pRb) in BCP-ALL patients’ cell extracts [25, 26, 29, 32]. The value of the finding stems from the widely accepted and central role that the sequential phosphorylation of pRb, or its family member proteins p107 and p130, has on the initiation of the G1/S transition [25, 26, 29, 32–39]. The aforementioned transition is regulated by the restriction point (RP) pathway [34]. However, several approaches about the details of this regulatory mechanism have been testified.

As presented in [40], two central theories on the biochemical regulation of the RP have been proposed. The first one constitutes the “current paradigm”, primarily trying to explain serum deprivation/re-stimulation experiments [34]. The second one is a newly proposed theory [40] (referred here as “new RP theory”) which is based on experimental data derived from cells being continuously exposed to growth factors.

In detail, according to the “current paradigm”, the stimulation of resting cells by growth factors leads to the progressive emergence of active Cyclin D:Cdk4,6 complexes. However, the “new RP theory” argues that Cyclin D:Cdk4,6 complexes are constitutively expressed and active throughout the cell cycle. Regarding the effects of these species on the RP execution, the “current paradigm” maintains that those effects lead to the partial inactivation of pRb, by hypo-phosphorylating it. This supported inactivation favors the expression of Cyclin E and the formulation of active Cyclin E:Cdk2 complexes as a result of the gradual liberation of the E2F transcription factors, which are crucial for the initiation of DNA replication, and Cyclin Dependent Kinase Inhibitor (CdkI) p27 sequestration, respectively. The emergence of active Cyclin E:Cdk2 complexes, finally, results in the terminal inactivation of pRb by hyper-phosphorylation. However, the hypo-phosphorylated form of pRb is increasingly reported to have growth suppression capabilities, primarily by suppressing E2F transcription factors [29, 37, 41]. Therefore, in the “new RP theory”, this finding is adopted and the “current paradigm’s” feedback loop is rejected. Regarding the activation of Cyclin E:Cdk2 complexes, the new theory involves a yet unknown activating modifier which activates Cdk2 by monitoring the metabolic input. This machinery is believed to function in a way similar to the yeast G1-phase activator Bck2. Moreover, according to this theory, Cyclin E:Cdk2 complexes are continuously expressed, but appear to be inactive during the early G1 and active in late G1 sub-phases. This activation pattern is shown to be in correlation with the oscillation of the active (hypo-phosphorylated) and the inactive (hyper-phosphorylated) forms of pRb respectively.

**Fig. 1:**
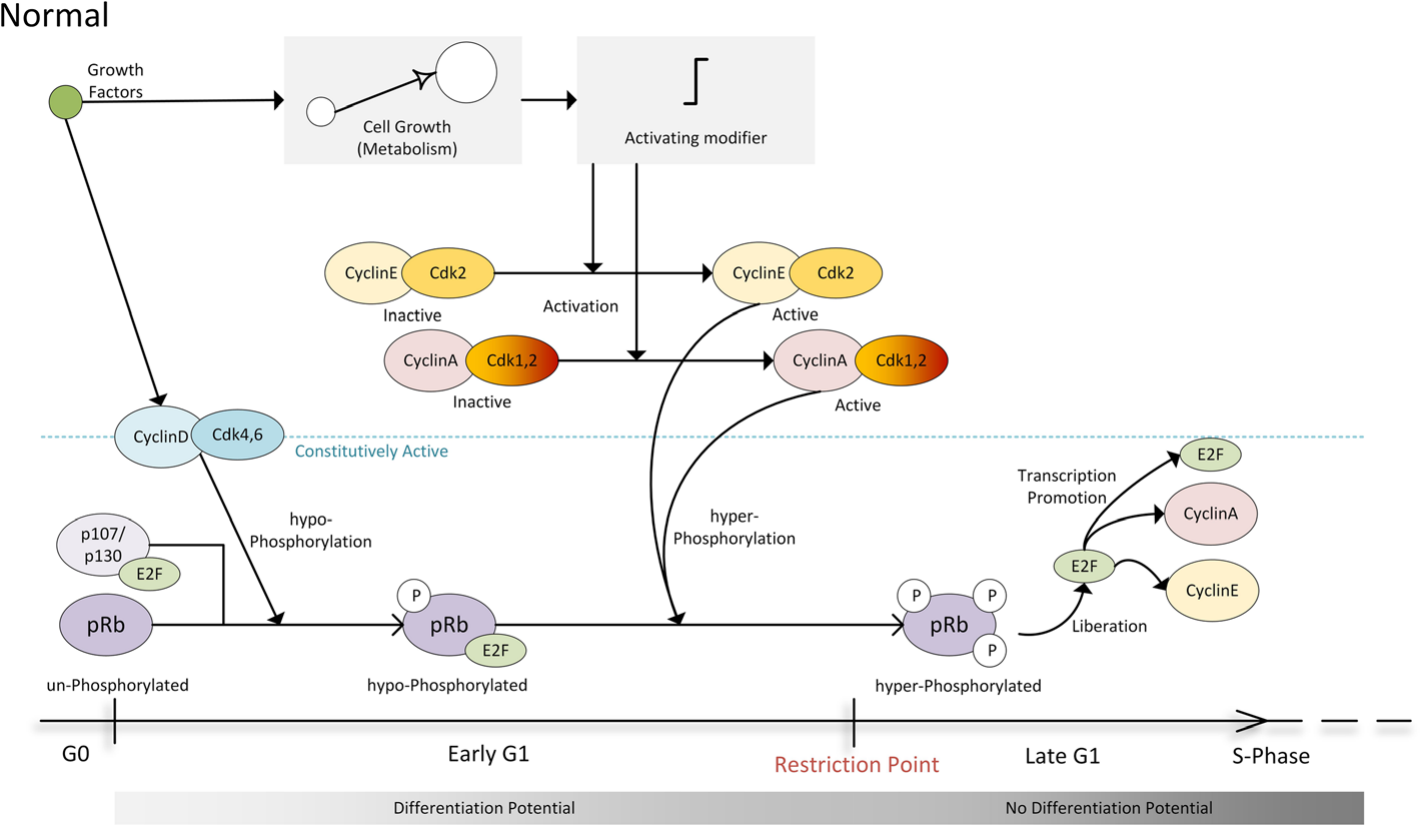
Main parts of the biochemical regulation of the G1/S restriction point in normal (non-leukemic) cell cycle. The presence of growth factors leads to the constitutive activation of Cyclin D:Cdk4,6 complexes, which in turn favors the hypo-phosphorylation of retinoblastoma protein (pRb) in early G1 phase. The hypo-phosphorylated pRb maintains the ability to inhibit E2F transcription factors. Growth factors also stimulate the metabolic machinery of the cell, leading its mass to gradually grow. When cell growth reaches a critical threshold, the Cyclin E:Cdk2 and Cyclin A:Cdk1,2 complexes are activated resulting in hyper-phosphorylation of pRb in late G1 phase (where the differentiation potential is lost), liberation of E2F transcription factors and increased Cyclin A (and Cyclin E, E2F) expression, whose levels are indicative of the passage to the S-phase

In order to validate the new RP theory, the authors of [40] have also developed a mathematical model in which the interference of metabolism in Cdk2 activation has been implemented by introducing a time-dependent switch machinery (activating modifier). This switch modifies the rates of Cdk2 activation-related reactions (switches them to non-zero value) after a predefined time interval related to cell growth rate. The basic principles of this theory (omitting details such as the inhibition of Cyclin:Cdk complexes by CdkIs for reasons of simplicity) are depicted in Fig. 1.

Based on what has been presented in previous paragraphs, the behavior of the cell cycle regulatory biochemical species in ALL cells seems to contradict the key principles of all the above theories about the RP. The hyper-phosphorylated status of pRb dictates an irreversible and rapid transition to the S phase. However, the transition rate is unexpectedly low and only a small percentage of leukemic cells is found in the S phase compared to their normal counterparts [24–26, 30, 32]. For example, in stimulated with cytokines CD34+ cells (i.e. cells expressing the hematopoietic progenitor cell antigen CD34), the hyper-phosphorylated form of pRb prevails and the percentages of cells in the S and G2/M phases are high (48.4 %).On the contrary, unstimulated CD34+ cells, in which only the un- and hypo-phosphorylated forms of the protein could be identified, mainly reside in G0/early-G1 phases [29]. This hyper-phosphorylated status of pRb may dictate that the vast majority of BCP-ALL cells are found specifically in the late-G1 phase of the cell cycle, at or beyond the restriction point. This state may explain their differentiation resistant phenotype [26]. Therefore, a deregulation in the G1 phase and the G1/S transition must have been caused in BCP-ALL cells due to their cancerous transformation.

In the process of identifying the molecular components that may be altered, it should be mentioned first that as far as the production and the activation of the Cyclin A are concerned, the G1/S transition is undisturbed [32]. This is unexpected, taking into account that the Cyclin A coding gene (*CCNA1*) is one of the known targets of E2F and the levels of the produced protein have been correlated with the passage of cells, including BCP-ALL cells, to the S-phase [32, 40]. Therefore, a deregulation that directly and uniquely refers to Cyclin A is excluded.

Looking at regulatory nodes upstream of the Cyclin A position in the cell cycle pathway, it has been shown that the sequential phosphorylation of pRb may be deregulated in BCP-ALL and that there is a limited redundancy between Cdk2 and Cdk4 in this phenomenon [26]. In more detail, it has been observed, both in NALM-6 and in ALL patient malignant cells, that the substrate specificities of Cdk4,6 are deregulated. This is evidenced by the finding that these kinases could also phosphorylate the serine 612 (ser612) phosphorylation site of pRb, which is generally believed to be Cdk2-preferred [26, 42–45]. Moreover, the hyper-phosphorylated version of the protein partially maintains its nuclear tethering and continues inhibiting E2F transcription factors (at least E2F-1) [25, 26, 32]. Notably, these phenomena occur to different extents among the sampled ALL patients, rendering them candidates to contribute to the observed inter-patient diversity in cell kinetics. A hypothesis formulated in [26] regarding the possible consequences of such a deregulation, refers to the possibility that the intervention of Cdk4,6 in Cdk2-preferred sites may lead to the creation of large phospho-groups in pRb before the involvement of Cdk2 (which is indicative of the passage to the late-G1 phase in normal cell cycle execution). Additionally, it has been reported [29] that in Western blot analysis experiments with ALL samples, multiple forms of pRb, between two and five, could be identified. Three of these forms, which show differential mobility on SDS-PAGE (Sodium Dodecyl Sulfate - PolyAcrylamide Gel Electrophoresis) depending on their phosphorylation level, may represent the un-, hypo- and hyper-phosphorylated statuses of the protein. However, one can speculate that at least one of the remaining reported forms might be the result of such a peculiar phosphorylation by Cdk4. This conjecture is in line with a hypothesis reported in literature [32], describing possible partial inactivation of pRb functions in ALL. This may lead to the loss of differentiation potential of BCP-ALL cells without, however, a commitment to complete the mitotic cycle and a traversal of the restriction point. A name is given to this alleged status of pRb as pseudo-hyper-phosphorylated pRb (pseudo-hyper-pRb). The identification of the possible consequences of deregulated sequential phosphorylation in the functionality of pRb could be characterized as a difficult task. Notwithstanding the extremely significant steps that have been taken towards unraveling the role of every phosphorylation site of pRb on the regulation of its function [41, 46–48], the entire picture is not yet fully uncovered. However, supporting our hypothesis, it has been shown in heterogeneous experiments that the phosphorylation of ser612 enhances or at least does not inhibit the aforementioned properties of pRb regarding E2F inhibition [49, 50].

During the preparation of the present study a new theory regarding the sequential phosphorylation of pRb by Cdk4 and Cdk2 [51] appeared. The authors present biochemical analyses of pRb protein in diverse cell lines. They show that Cdk4 in early G1 phase, instead of progressively phosphorylating multiple preferred sites and therefore leading pRb to the hypo-phosphorylation status, it mono-phosphorylates one and only one of fourteen different sites (including those believed to be Cdk2-preferred) in each instance of the protein. During the passage to late-G1 phase, Cdk2 completely hyper-phosphorylates the mono-phosphorylated pRb isoforms in a quantum switch-like step (>12 phosphates). However, such a theory seems to contradict the finding in non-malignant stimulated CD34+ cells in which ser612 is not found to be phosphorylated by Cdk4 [26]. Moreover, the plurality of pRb forms reported in western blot experiments from ALL patient samples [29] cannot be explained by this theory.

**Fig. 2:**
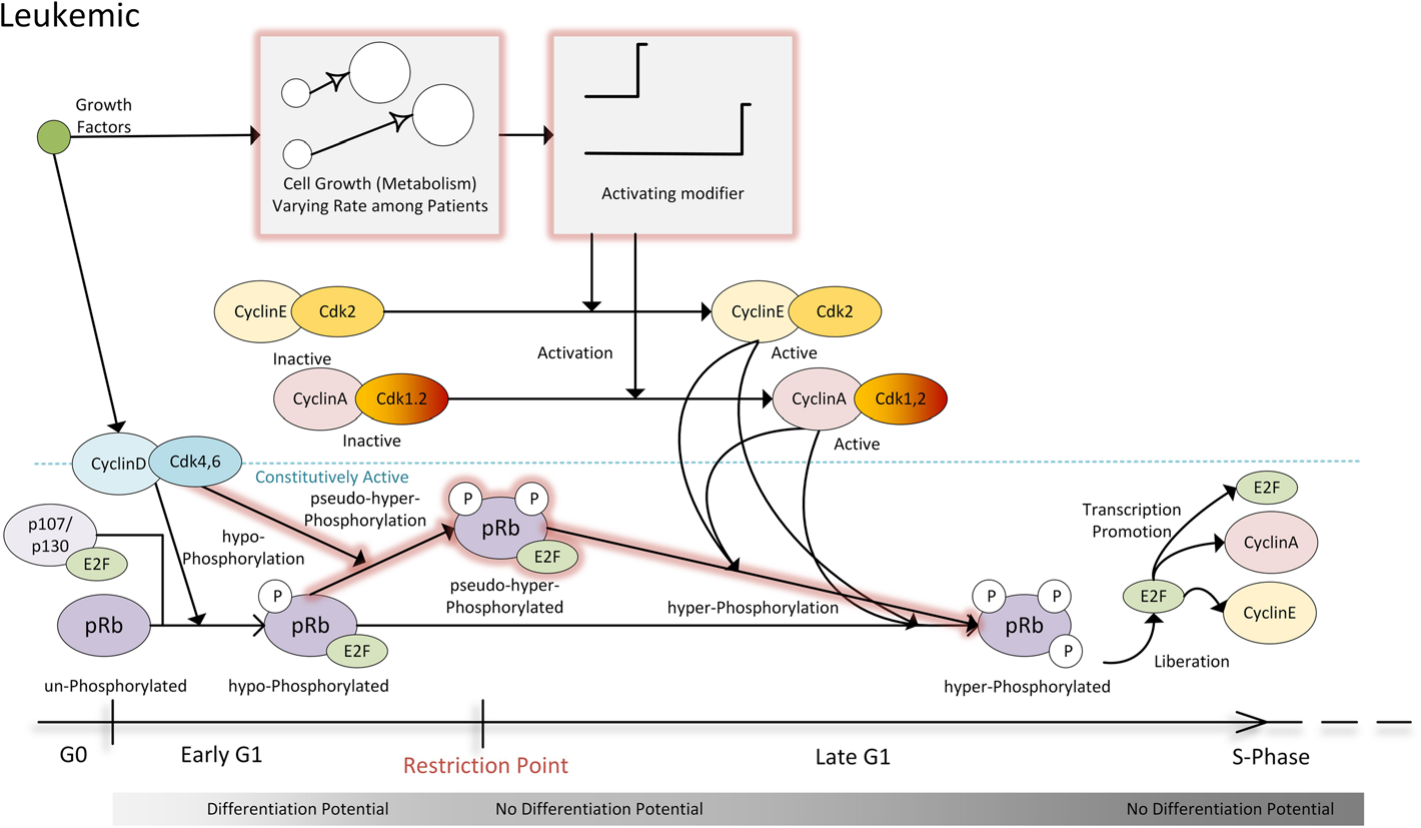
G1/S restriction point alterations and deregulations in BCP-ALL. In contrast with the normal cell cycle pathway (Fig. 1), Cyclin D:Cdk4,6 complexes except for hypo-phosphorylating pRb may also lead the protein to an intermediate phosphorylation status (termed “pseudo-hyper-phosphorylated”) which retains the ability to inhibit E2F transcription factors, although its phosphorus content is increased. This version of the protein is believed to have lost differentiation related functions [32], therefore its accumulation implies that the cell resides at or beyond the restriction point. Only when Cyclin E:Cdk2 and Cyclin A:Cdk1,2 complexes are activated, could the hypo-phosphorylated and pseudo-hyper-phosphorylated versions of the protein become hyper-phosphorylated and consequently liberate E2F transcription factors. The metabolism-mediated activation of these complexes is believed to exhibit differential time-course among patients due to differences in metabolism rates

Taking into account all the aforementioned findings, the central deregulations thought to be important in the execution of the restriction point in leukemic cells are presented in Fig. 2.

The main differences (highlighted in red) between what is presented in Fig. 1 and the altered situation presented in Fig. 2, concerns the ability of Cyclin D:Cdk4,6 complexes to also autonomously pseudo-hyper-phosphorylate pRb. Based on the “new RP theory”, these complexes are believed to be always active during the whole G1 phase (in contrast with Cyclin E:Cdk2). Therefore they may contribute to the creation of higher phosphorylated forms of the protein from the very first hours of the execution of the cell cycle. The latter could explain the almost completely dominant hyper-phosphorylated status of pRb in ALL patients’ cells. However, the complete liberation of E2F from pRb is not allowed until pRb is terminally hyper-phosphorylated. This could be done either after its pseudo-hyper-phosphorylation or directly from the hypo-phosphorylated state by Cdk2. As shown in [25], Cdk2 is also active and able to phosphorylate ser612 [26] in BCP-ALL cells.

Finally, significant differences in the glucose metabolism rates have been reported between Prednisone Sensitive and Prednisone Resistant ALL patients [27] (Prednisone is a glucocorticoid drug that is included in the main core of the ALL treatment [52, 53]). Therefore, the varying rate of metabolism, through the action of the activating modifier, that may lead to differential regulation of Cyclin E:Cdk2 complexes activation (delayed or accelerated), is thought to be crucial in order for the observed diversity in the leukemic cell cycle kinetics to be explained.

The aforementioned description of the biochemical regulation of the cell cycle in BCP-ALL opposes even the generally accepted biochemical dynamic trends for the G1 phase restriction point. Thus, its direct simulation by existing models [35, 40, 54–60] (e.g. by simply altering the parameter values for a number of kinetic rate constants) is thought not to be feasible. For this reason, the necessity for the creation of a new model has arisen. However, the possibility to use one of these models as the basis for the development of the newly proposed model has been investigated by formulating concrete criteria as presented in the Results and discussion and in Methods sections.

In this context, the central target of the present study has been primarily to investigate if the introduction of the above presented deregulations into an already established cell cycle model by modifying its structure and recalibrating its parameters is capable to alter its behavior so as to simulate the sub-cellular dynamics and cell cycle kinetics observed in BCP-ALL cells. Additionally, a successful adaptation of the model may render it an essential component of a personalized predictor for the *T*_*c*_ value. The latter could take place following pertinent input perturbation based on experimental and clinical findings about ALL. The steps followed in order for the newly proposed model to be defined are given in detail in the following sections.

## Results and discussion

### Reference model selection and simulation

Based on specific criteria, presented in detail in the Methods section, the model chosen to form the basis for the newly proposed model has been the one described in [40], hereafter referred to as the “reference model”.

**Fig. 3:**
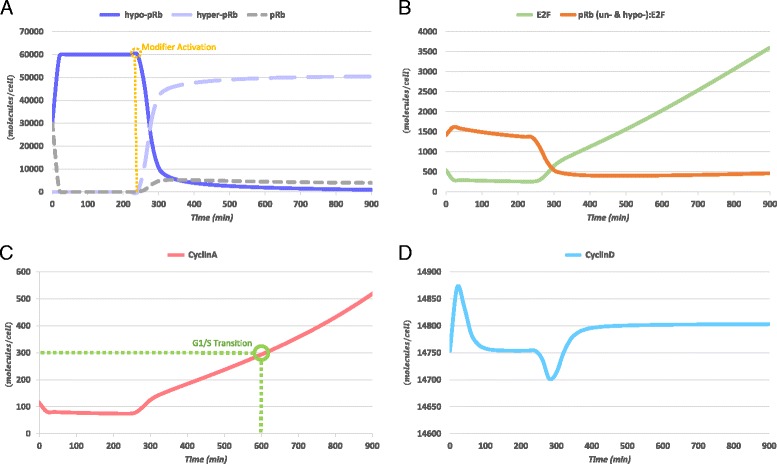
Simulation results of the reference model [40] for 900 *min* (15 *h*). Human HCT116 colon carcinoma cells grown under conditions of constant growth factor exposure. (**a**) The levels of hypo-phosphorylated pRb (hypo-pRb, purple) rapidly rise during the first *min* of G1 phase, due to phosphorylation of un-phosphorylated pRb species (pRb, grey) by CyclinD:Cdk4,6, and remain steadily high until the activation of the metabolism-related activating modifier switch (at 240 *min*); this in turn activates Cyclin E:Cdk2 and Cyclin A:Cdk1,2 complexes (Cyclin E:Cdk2 not shown in the figure). As a consequence, the majority of hypo-pRb is transformed to hyper-phosphorylated pRb (hyper-pRb, light purple). (**b**) In the time interval during which the levels of pRb and hypo-pRb are significant, free E2F transcription factors (E2F, green) are predominantly bound to these versions of pRb (orange). When hyper-pRb starts to dominate the levels of retinoblastoma protein, E2F is liberated and the levels of free transcription factors quickly elevate. (**c**) Cyclin A levels (red) show steady or even decreasing trends until the activation of the modifier switch. After this activation they gradually rise, due to E2F liberation, reaching the indicative of S-phase passage 300 (*molecules/cell*) threshold at approximately 600 *min* (indicated in green). (**d**) Cyclin D levels (cyan) do not show any significant variation during the execution of the model

In Fig. 3 the simulation results of this model in its original version [40] are presented in order to be easily and directly compared with the results acquired after the BCP-ALL-related modifications.

As can be seen in Fig. 3a the hypo-phosphorylated form of pRb clearly dominates the levels of this protein for about the first 300 min (*min*) of the simulation. Moreover, for the same time range, the levels of E2F transcription factors and Cyclin A (Fig. 3b and c respectively), are not showing any increasing trends due to the inhibition by pRb (Fig. 3b). However, when the levels of hyper-pRb start to rise, which is a consequence of the activation of the modifier switch (at 240 *min*), E2F and Cyclin A levels rapidly start to elevate. This is a more or less typical behavior of a model simulating the dynamics of the restriction point of the cell cycle which is not encountered in BCP-ALL cells. The steps taken in order to construct a model able to simulate the altered dynamics are described in the following sections.

### Modifications leading to the proposed model

**Fig. 4:**
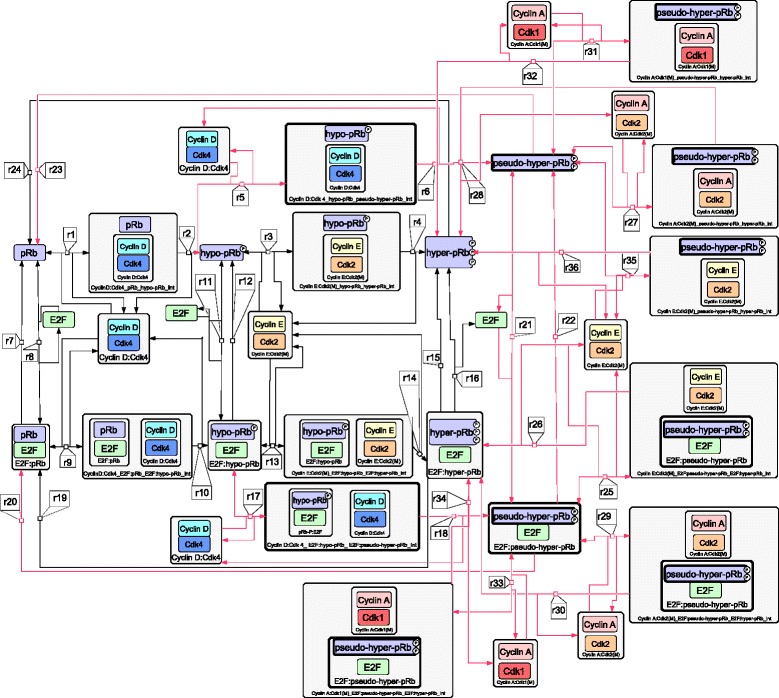
Part of the structure of the newly proposed model in SBGN format (only modified regions in relation to the reference model of [40]). The newly introduced species are encircled by a thicker frame and the new reaction arrows are colored red. The (M) addition appended after the species/complexes names indicates activation by the activating modifier

Based on the previously presented deregulations in BCP-ALL cell cycle sub-cellular biochemical dynamics, specific additions and modifications have been made in the reference model, concerning both its structure (biochemical reactions) and its parameters values (reaction rates). The complete set of molecular species and reactions of the reference model, together with their newly introduced counterparts are given in Additional file 1: Table S1. A detailed description of every new reaction and biochemical species is given in the following paragraphs. Moreover, they are presented in Systems Biology Graphical Notation (SBGN) format [61], together with the parts of the reference model that are directly related to them, in Fig. 4.

In total, 10 new molecular entities and 29 new reactions have been added to the 49 species and 138 reactions of the reference model. As shown in Fig. 4, the retinoblastoma protein can be found in four distinct phosphorylation states, namely the un-phosphorylated (pRb), the hypo-phosphorylated (hypo-pRb), the hyper-phosphorylated (hyper-pRb) and the pseudo-hyper-phosphorylated (pseudo-pRb-PP) states. Similarly to the reference model, the un-phosphorylated form plays also the role of p130 and p107. The first three states are inherited from the reference model, whereas the last one is defined *de novo* in the present study. In order for the transitions among them to be realized, intermediate complexes are formed between pRb, hypo-pRb or pseudo-hyper-pRb and Cyclin D:Cdk4,6, Cyclin E:Cdk2, Cyclin A:Cdk2 or Cyclin A:Cdk1 complexes (Reactions: r1,r3,r5,r23,r27,r31). However, the transitions to the next state (Reactions: r2, r4, r6, r36, r28, r32) are partially realized and a percentage of complexes disassociate by leaving the pRb, hypo-pRb or pseudo-hyper-pRb to their previous state (reverse parts of reactions: r1, r3, r5, r35, r27, r31). Similarly and in parallel, the transition among the different phosphorylation states can also be done when E2F is bound to the different forms of pRb protein (Reactions: r9, 13, r17, r25, r29, r33 and r10, r14, r18, r26, r30, r34).

In the model part adopted from the reference model, the pRb and hypo-pRb forms can bind to the E2F transcription factors by a simple reaction (r8, r11), forming the pRb:E2F and hypo-pRb:E2F complexes. On the contrary, the hyper-pRb form cannot bind de novo to the E2F, and any E2F species already bound to hyper-pRb (hyper-pRb:E2F complexes) before the hyper-phosphorylation step can only be liberated (r16). This liberation step could also be realized for pRb and hypo-pRb (reverse part of r8, r11). However, as previously mentioned, these forms of the protein could re-bind E2F. For the case of pseudo-hyper-pRb, this version of the protein can de novo inhibit free E2F forming the pseudo-hyper:pRb:E2F complexes (r21). Finally, E2F bound to any form of pRb can be directly degraded (Reactions: r7, r12, r15, r22).

Regarding the mathematical definition of the newly introduced parts of the model, mass action kinetic laws have been adopted in a way similar to the original study. Additionally, these new reactions are in general modified versions of existing ones in the reference model. To that end a linear relationship between their reaction rates and those of their corresponding reactions has been assumed and implemented by the addition of a proportionality constant. These relationships are presented in Table 1.

**Table 1:** Mathematical definition of the reaction rate parameters of the newly introduced reactions

Relevant original reaction number in Fig. 4	Newly introduced reaction number in Fig. 4	Parameter name in original reaction^a^	New parameter definition^b^
r15	r22	*k*_*dE*2*F*_	${k}_{dE2{F}_{leuk}}=p{r}_{dE2F}*{k}_{dE2F}$
r1	r5	*k*_*bD*4*pRb*_	${k}_{bD4 pRbleuk}=p{r}_{bD4}*{k}_{bD4pRb}$
r9	r17	*″*	*″*
r1 (reverse)	r5 (reverse)	*k*_*uD*4*pRb*_	${k}_{uD4 pRbleuk}=p{r}_{uD4}*{k}_{uD4pRb}$
r9 (reverse)	r17 (reverse)	*″*	*″*
r2	r6	*k*_*upD*4*pRb*_	${k}_{uD4 pRbleuk}=p{r}_{uD4}*{k}_{uD4pRb}$
r10	r18	*″*	*″*
r24	r23	*k*_*tpRbDephos*_	${k}_{tpRbDepho{s}_{leuk}}=p{r}_{tpRbDepho s}*{k}_{tpRbDepho s}$
r19	r20	*″*	*″*
r16	r21	*k*_*uE*2*FpRb*_	${k}_{uE2 FpRb\_ leuk}=p{r}_{uE2F}*{k}_{uE2 FpRb}$
r11	r21 (reverse)	*k*_*bE*2*FpRb*_	${k}_{bE2 FpRb\_ leuk}=p{r}_{bE2F}*{k}_{bE2 FpRb}$
r3	r35	*k*_*bE*2*pRb*_	${k}_{bE2pR{b}_{leuk}}=p{r}_{bE2A2A1}*{k}_{bE2pRb}$
r13	r25	*″*	*″*
n.s.	r27	*k*_*bA*2*pRb*_	${k}_{bA2pR{b}_{leuk}}=p{r}_{bE2A2A1}*{k}_{bA2pRb}$
n.s.	r29	*″*	*″*
n.s.	r31	*k*_*bA*1*pRb*_	${k}_{bA1pR{b}_{leuk}}=p{r}_{bE2A2A1}*{k}_{bA1pRb}$
n.s.	r33	*″*	*″*
r3 (reverse)	r35 (reverse)	*k*_*uE*2*pRb*_	${k}_{uE2pR{b}_{leuk}}=p{r}_{uE2A2A1}*{k}_{uE2pRb}$
r13 (reverse)	r25 (reverse)	*″*	*″*
n.s.	r27 (reverse)	*k*_*uA*2*pRb*_	${k}_{uA2pR{b}_{leuk}}=p{r}_{uE2A2A1}*{k}_{uA2pRb}$
n.s.	r29 (reverse)	*″*	*″*
n.s.	r31 (reverse)	*k*_*uA*1*pRb*_	${k}_{uA1pR{b}_{leuk}}=p{r}_{uE2A2A1}*{k}_{uA1pRb}$
n.s.	r33 (reverse)	*″*	*″*
r4	r36	*k*_*upE*2*pRb*_	${k}_{upE2pR{b}_{leuk}}=p{r}_{upE2A2A1}*{k}_{upE2pRb}$
r14	r26	*″*	*″*
n.s.	r28	*k*_*upA*2*pRb*_	${k}_{upA2pR{b}_{leuk}}=p{r}_{upE2A2A1}*{k}_{upA2pRb}$
n.s.	r30	*″*	*″*
n.s.	r32	*k*_*upA*1*pRb*_	${k}_{upA1pR{b}_{leuk}}=p{r}_{upE2A2A1}*{k}_{upA1pRb}$
n.s.	r34	*″*	*″*

n.s.: not shown in Fig. 4

^a^The parameter names for the already defined reaction rates are kept as introduced in [40]. ^b^The respective newly introduced parameters are similarly named but denoted by the *leuk* subscript. The proportionality constants are named using the symbol *pr* and a subscript influenced by their relevant reaction rate name

Having introduced the new reactions and species into the model by using the SBML [62] compatible tool COPASI [63] in a way explained in detail in Methods, the next steps of the analysis refer to the estimation of the kinetic parameters of the augmented model.

### Model calibration objectives

**Fig. 5:**
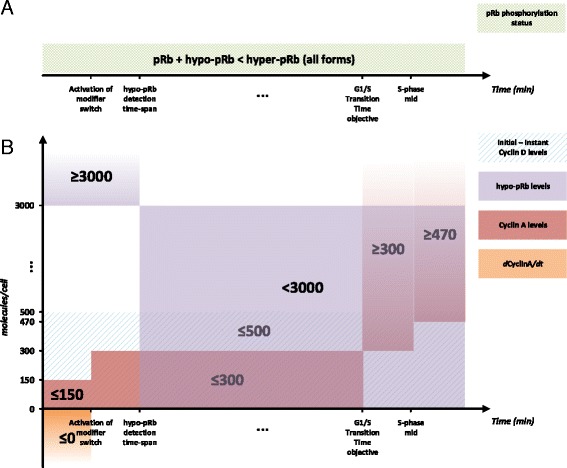
Semi-quantitative/qualitative criteria used for the calibration of the newly proposed model by optimization. The criteria are defined for (**a**) the phosphorylation status of the different pRb forms and (**b**) the levels of central model species

Before calibrating the model, the objectives for this procedure have to be set concerning the dynamics of the system. These are mainly defined in terms of semi-quantitative/qualitative criteria for the dynamic levels of species due to the lack of time course and quantitative data for the levels of molecular entities modeled. The criteria are illustrated in Fig. 5 and a detailed listing, together with the related quantitative information and the reference sources for them are given in Table 2. A thorough description of the criteria derivation process is given in Additional file 2. However, a brief discussion is provided in the following paragraphs. Finally, the formulated criteria are translated into objective function components and optimization constraints as presented in the subsequent sections.

**Table 2:** (Semi-) Quantitative information extracted from literature used for the calibration of the model

Property		Value	Source
1.pRb phosphorylation levels			
1.a	hyper-pRb domination in BCP-ALL cells	qualitative	ALL-specific: [29]
1.b	hypo-pRb positive BCP-ALL cells	16 % (±13.2 %)	*″*
1.c	hypo-pRb detection threshold	3000 (*molecules/cell*)	Generic: derived by reference model simulation ([40] and Additional file 2)
2.Cell Kinetics			
2.a	Cycling cells	94 %	ALL-specific: [29]
2.b	Quiescent cells	2 %	ALL-specific: [28, 29]
2.c	Apoptotic cells	4 %	ALL-specific: [30]
2.d	Cycling cells in S/G2/M phases	10 %	ALL-specific: [25–27]
2.e	Cycling cells in G1 phase	84 %	ALL-specific: Derived from 2.a-d (see Additional file 2)
2.f	Cycling cells in early-G1	13.7 %	ALL-specific: Derived from 1.b. and 2.a. (see Additional file 2)
3.Cell cycle Timings			
3.a	Mean Cell cycle duration of BCP-ALL cells (*T*_*c*_)	112.5 *h* (±46.8 *h*)^a^	ALL-specific: [24]
3.b	S-phase duration in BCP-ALL cells (*T*_*s*_)	18 *h*	*″*
3.c	G2-phase duration	6 *h*	Generic: [74]
3.d	M-phase duration	1 *h*	*″*
3.e	Mean G1-phase duration in BCP-ALL cells	87.5 *h* (±46.8 *h*)	Derived from 3.a-d (see Additional file 2)
	Estimated realistic time-range for G1/S transition in BCP-ALL	~2400 - ~8000 *min*	*″*
3.f.	early-G1 phase duration in BCP-ALL cells	~800 *min*	Derived from 2.f and 3.e (see Additional file 2)
3.g.	S-phase midpoint	9 *h* after G1/S transition	ALL-specific (see Additional file 2)
4.Cyclin A levels			
4.a	S-phase passage levels	300 (*molecules/cell*)	Generic: derived by reference model simulation ([40] and Additional file 2)
4.b	Levels (rate) before activating modifier activation	<150 (*molecules/cell*) (<=0 ((*molecules/cell*)/*min*))	*″*
4.c	Levels after activating modifier activation	<300 (*molecules/cell*)	*″*
4.d	Levels at the midpoint of S-phase	470 (*molecules/cell*)	*″*
5.Cyclin D levels			
	Maximum difference between the initial and the instant levels	500 (*molecules/cell*)	Generic: derived by reference model simulation [40]

^a^Mean (±standard deviation)

In general, the model should at the same time predict a behavior of Cyclin A and E2F similar to the one of the reference model, yet widened in time due to delayed G1/S transition. On the contrary, for the hypo- and the hyper-phosphorylated forms of pRb the behavior should be reversed in the first hours of the G1 phase execution where the early G1 phase would be normally present.

In this context, there are 5 categories of criteria to be met. Starting from the pRb phosphorylation status (Fig. 5a and properties set 1 in Table 2), the hyper-phosphorylated forms of pRb (pseudo-hyper-pRb and hyper-pRb) should generally dominate the total levels of this protein. However, its hypo-phosphorylated counterpart could also be detected in BCP-ALL cells (Fig. 5b and 1.b in Table 2). The existence of hypo-pRb is indicative of the presence of cells in early G1-phase. Therefore, one can speculate that leukemic cells also exhibit an early-G1-like phase during the first hours of their cell cycle. In this context, during the calibration of the model, specific constraints have been set in order for hypo-pRb to be at least higher than a detection threshold (1.c in Table 2) for a pre-specified time span (hypo-pRb detection time span shown in Fig. 5b and 3.f in Table 2). This has been done under the assumption that hypo-pRb becomes undetectable when cells are leaving the early-G1 phase.

It should be especially mentioned that although the detection level thresholds defined in Table 2 have been formulated based on experiments conducted on a different cell type (colon carcinoma cell line) [40], it is believed that these thresholds constitute a realistic approximation for the detectable levels of the in focus proteins in any similar experiment.

The BCP-ALL cell kinetics (properties set 2 of Table 2) has been extracted from the related literature in order to assist on the estimation of any missing Cell Cycle timings (properties set 3 in Table 2) that the model should reproduce after its calibration. Among these timings, those of special interest are the Mean G1-phase duration (3.e in Table 2), a realistic range for the duration of this phase and the early-G1 phase duration (3.f in Table 2). The last property is used as a temporal threshold for the existence of detectable hypo-pRb levels while the first and second properties determine the time point in which the S-phase transition is realized. Therefore these properties play a central role in the calibration of the model. Given that Cyclin A levels are correlated with the percentage of BCP-ALL cells in S-Phase [32], the criterion for the G1/S transition has been set to the increase of Cyclin A to specific levels (4a in Table 2). Therefore, in order to accept that a given parameterization of the model predicts a specific duration of the cell cycle, the levels of Cyclin A should reach this predefined threshold within the expected G1/S transition time (G1/S Transition Time objective in Fig. 5b). Moreover, by knowing that the G1/S transition is found undisturbed regarding the Cyclin A-related phenomena [32], specific criteria have to be set in order to ensure that the trends of Cyclin A dynamics remain unchanged compared to those in the reference model (properties set 4 in Table 2 and Additional file 2).

The last objective set for the calibration of the model concerns the levels of Cyclin D. Their low fluctuation trends are shown to be a central characteristic of the analysis done during the development of the reference model [40]. Therefore, a criterion has been set for the maximum difference between the initial and the instant levels of Cyclin D not to exceed the 500 (*molecules/cell*) threshold. The choice of this value has been influenced by the dynamical behavior of the protein in the reference model (Fig. 3).

The introduction of all the above criteria into the model in terms of implementation is presented in detail in Methods.

### Calibration of the model

The calibration of the model has been performed using the optimization functionality in COPASI. For the assessment of the fulfillment of the calibration criteria, an objective function has been defined following the linear scalarization method for the multi-objective optimization. A detailed description of the definition and implementation of the objective function is given in Methods. Briefly, for every time point in which a criterion is not fulfilled, a unitary penalty point is added to the overall sum of penalties for a simulation time course concerning a specific parameterization of the model. The Particle Swarm optimization algorithm [64], available in COPASI, has been used in order for the objective function to be minimized. This has been done by tuning a number of model parameters, as presented in the following paragraphs.

First, the majority of the newly introduced proportionality constants in Table 1 have been chosen to be tuned. An exemption has been made for *pr*_*dE2F*_ since there is no evidence, to the extent of our knowledge, for an altered E2F degradation in BCP-ALL. Therefore its value has been fixed at 1. Similarly *pr*_*uD4*_ and *pr*_*uE2A2A*1_ values have also been fixed at a unitary value since *k*_*uD4pRb*_ and *k*_*uE2pRb*_ have also been set to be constant in the reference model. The value ranges of the parameters to be tuned during the parameter estimation procedure are given in Table 3. In general, partial existence of the aberrant phenomena should be acquired. Therefore, the proportionality constants have been allowed to vary in the range [0,1]. Moreover, for the case of *pr*_*uE*2*F*_ and *pr*_*tpRbDephos*_, only values lower than one (or at least equal to) may explain the continuum inhibition of E2F by pRb even in pseudo-hyper-phosphorylated state and take into account the hypothesis for possibly deregulated de-phosphorylation of pRb in BCP-ALL [25]. However, it cannot be excluded that the significant inhibition of Cyclin A expression (as dictated by the delayed G1/S transition) is a consequence of the not only existing but enhanced ability of pseudo-hyper-phosphorylated pRb to de novo bind E2F. Therefore, an exception has been made for *pr*_*bE*2*F*_, which has ranged in [0,2].

**Table 3:** Model calibration results

Parameter name	Range in parameter space	Estimated value
*pr*_*bD*4_	[0–1]	0.841699
*pr*_*upD*4_	[0–1]	0.484118
*pr*_*tpRbDephos*_	[0–1]	1
*pr*_*uE*2*F*_	[0–1]	0.612768
*pr*_*bE*2*F*_	[0–2]	1.88407
*pr*_*bE*2*A*2*A*1_	[0–1]	0.130145
*pr*_*upE*2*A*2*A*1_	[0–1]	0.428204
*ModifierTime*	120–780 *min*	675.993 *min*
*K*_*sCyclinD*_	100–2000 ((*molecules/cell*)*/min*)	1761.08 ((*molecules/cell*)*/min*)
Cyclin D Initial Levels	10000–20000 (*molecules/cell*)	19264 (*molecules/cell*)

Regarding the *ModifierTime* parameter, in the original version of the model the activation of this switch takes place at the end of early G1. The specific time point has been estimated based on experimental data. However, in the case of BCP-ALL, normal early-G1 is omitted/shortened, the duration of the G1 phase is prolonged and glycose metabolism is altered and varying among patients. Subsequently, we hypothesize that the point of this activation may be different from the value adopted in the reference model. Therefore, this parameter has been considered a candidate for changes during the calibration of the model. Moreover, by inspecting the reference and the newly proposed model, the *ModifierTime* parameter has been found to primarily determine the time point after which hypo-pRb becomes undetectable. Thus, it is believed that the value of the parameter should be smaller than the estimated early-G1-like phase duration. In this context, the range for this parameter has been set to [120, *hypo-pRb detection time span*) *min*, which for the mean case could be translated to [120,800) *min*, as previously described. For the needs of the optimization procedure the range has been slightly changed as presented in Table 3, since the *ModifierTime* parameter should be smaller than 800 *min*. Additionally, the omission/shortening of early-G1 phase in ALL may dictate the activation of the activating modifier even earlier than in the reference model case. This explains the choice of a relatively low lower limit which is considered necessary in order to search an adequately large area in the parameter space.

In order to render the model able to fulfil the Cyclin D related criterion, the rate of Cyclin D production (*k*_*sCyclinD*_) together with the initial levels of this protein have been re-estimated. Another justification for choosing these parameters could be given by the observation that among BCP-ALL patients Cyclin D is differentially expressed [25].

Finally, regarding the initial levels of the different forms of pRb, it has been decided - in contrast with the reference model (where a synchronization of cells in early-G1 was followed) - to initiate the model with the existence of only un-phosphorylated forms of pRb. By taking into account that pRb is dephosphorylated after mitosis [65, 66] this may enable the model to simulate the whole time span of G1-phase. Therefore, the available pool of pRb and pRb:E2F species has been distributed exclusively in un-phosphorylated species (Table 2 of Additional file 1). The initial levels of the other species have remained unchanged.

### Calibration results and model testing

By executing the global stochastic optimization procedure (see Methods), a set of estimations for the tunable model parameters has been acquired as shown in Table 3.

**Fig. 6:**
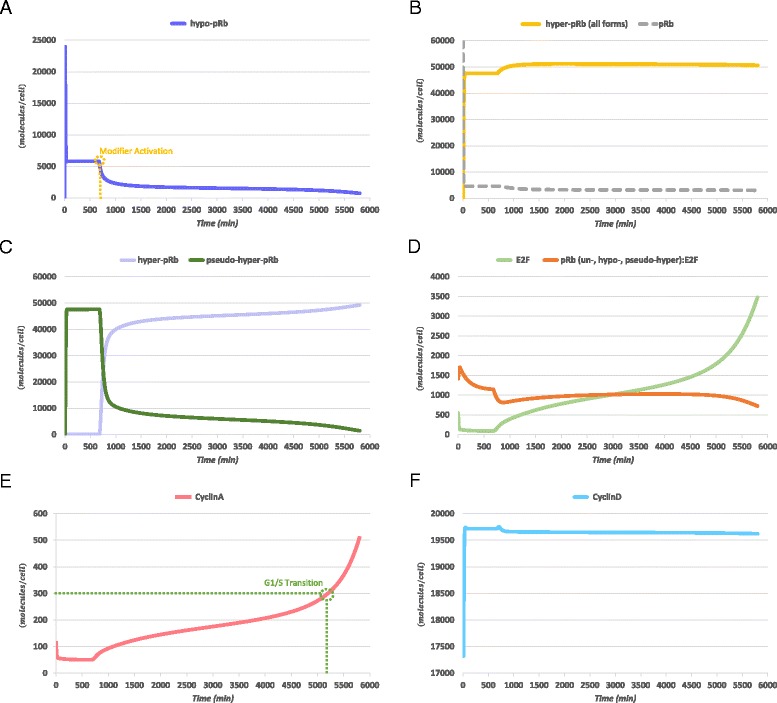
Simulation results of the newly proposed model after estimating its parameters for the mean case. (**a**) Hypo-phosphorylated retinoblastoma protein (hypo-pRb, purple) although rapidly formatted at the start of the G1-phase, maintains significant levels only for a limited period of time. (**b**) Hyper-phosphorylated forms of retinoblastoma protein (hyper-pRb all forms, dark yellow) rapidly dominate the total levels of the protein in contrast with the un-phosphorylated form (pRb, grey) which is quickly consumed. (**c**) Pseudo-hyper-phosphorylated retinoblastoma protein (pseudo-hyper-pRb, dark green) is directly formulated from the very first hours of the G1-phase and exclusively represents the hyper-phosphorylated forms of the retinoblastoma protein until the Modifier Activation time point, after which hyper-phosphorylated retinoblastoma (hyper-pRb, light purple) prevails. (**d**) Significant free E2F levels (E2F, green) are appointed only after the Modifier Activation. However, adequate levels of E2F are bound to E2F inhibiting pRb forms (orange) for a substantial time interval. (**e**) Cyclin A levels behavior (red) is consistent with the criteria set, showing decreasing or steady trends for the first hours of the simulation and increasing ones till its end, reaching the G1/S Transition threshold in 5200 *min*. (**f**) Cyclin D (light blue) shows insignificant variation in its levels for the entire time course of the simulation

The simulation results, for 6000 *min* (several hours beyond the G1/S Transition Time objective for the mean case) using this parameter set are shown in Fig. 6.

As can be observed in Figs. 6a and b, the hyper-phosphorylated forms clearly dominate the levels of the pRb protein in contrast with the original version of the model (Fig. 3). Moreover, hypo-pRb (Fig. 6a) levels are significant only for a limited period of time in agreement with the criterion set for the duration of the early-G1-like phase. An interesting result is derived from the levels of pseudo-hyper-pRb and hyper-pRb species (Fig. 6c). When these levels are compared to those of hypo-pRb and hyper-pRb in Fig. 3, an analogous picture is encountered, but hypo-pRb is now substituted by pseudo-hyper-pRb. However, due to the preservation of the ability of pseudo-hyper-pRb to inhibit E2F (Fig. 6d) and although hyper-pRb is immediately and abundantly expressed after the activation of the activating modifier switch, the expression of Cyclin A (Fig. 6e) exhibits significantly lower rate, at least for the first 4000 *min* (~65 *h*). Only when significant levels of E2F are liberated does Cyclin A show noticeably increasing trends that indicate and favor passage to S-Phase.

Thus, the model predictions are believed to be consistent with the observed cell cycle dynamics in BCP-ALL, and with the mean temporal dynamics for the passage to the S-phase. Importantly, a conclusion that could be extracted is the following. The restriction point machinery is recalibrated in such a way that the inhibition of E2F transcription factors is now mediated by a more phosphorylated form of pRb. Nevertheless, this inhibition takes place for a significant amount of time in a way similar to the one observed in the normal execution of the restriction point. Most importantly, the role of un- and hypo-phosphorylated versions of the protein that may favor the maturational progress of the cell is almost omitted.

### Hypothesis testing

The final step of the model analysis has been to test it for a number of additional scenarios and hypotheses aiming at investigating its conformity with additional findings and demonstrating its potential applicability as a predictive tool.

**Fig. 7:**
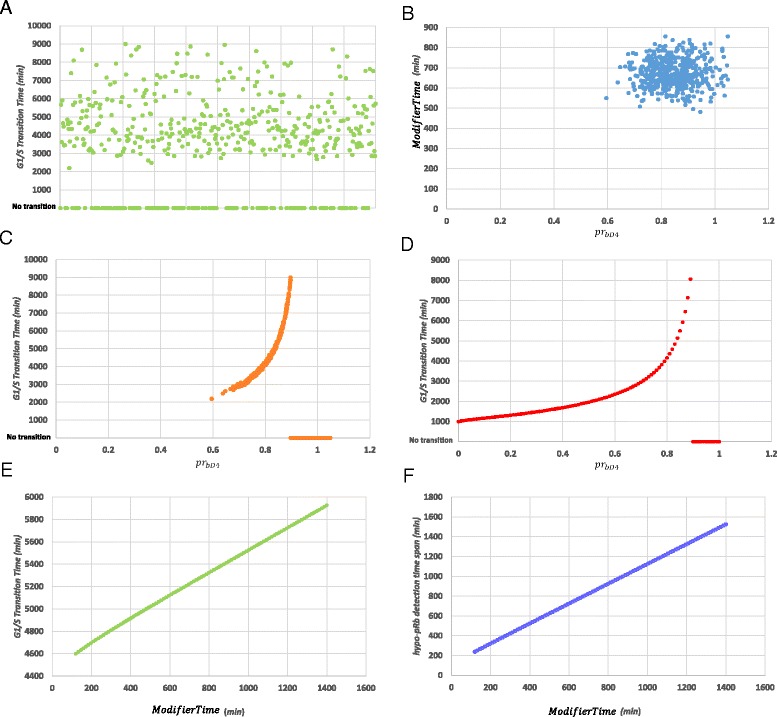
Random sampling and parameter scan result for *pr*_*bD*4_ and *Modifier Time* parameters. (**a**) By randomly sampling the two parameters, the model predicts a wide range of values for the G1/S Transition Time-point. (**b**) Parameter value samples taken during the random sampling procedure (**c**) Relationship between the observed G1/S Transition Time and *pr*_*bD*4_ values in random sampling parameter scan (**d**) Relationship between the observed G1/S Transition Time and *pr*_*bD*4_ values for constant value of *Modifier Time* (**e**) Relationship between G1/S Transition Time and *Modifier Time* for constant value of *pr*_*bD*4_ (**f**) Relationship between hypo-pRb detection time span and *Modifier Time* for constant value of *pr*_*bD*4_

As a first step, a parameter scan procedure has been followed using the corresponding functionality in COPASI. The objectives for this procedure have been to identify whether the model is able to predict that for BCP-ALL the G1/S transition will happen inside a realistic time range after perturbing a set of its parameters (Table 2) and to explain the observed significantly larger standard deviation of *T*_*c*_ in BCP-ALL. The parameters chosen to be perturbed have been the *pr*_*bD*4_ proportionality constant and the *ModifierTime*. As mentioned in Background, these parameters are related to two phenomena which according to literature vary among ALL patients. The first one refers to the extent to which Cdk4 contributes to the pseudo-hyper-phosphorylation of pRb, whereas the second one refers to the metabolic rate of the cell. 500 random sampling steps have been executed using two normal distributions, one for each aforementioned parameter. Their mean values have been set to the estimated parameter values during the calibration step (Table 3) and their standard deviations to the 10 % of the mean values respectively. For every sampling step the model has been simulated for 9000 *min* (150 *h*), that means several hours beyond the maximum G1-phase duration in the estimated range (Table 2). The results of this procedure are shown in Fig. 7.

As can be observed in Fig. 7a, by randomly sampling the parameter values the transition to S-phase is predicted to take place in a wide range of time points which sufficiently meets the value range presented in Table 2. The samples taken from the two-dimensional parameter space are given in Fig. 7b. For a number of simulations, however, a failure to proceed to the S-phase, at least before the 9000 *min* threshold, is predicted. The latter is indicated by a zero G1/S Transition Time value. This is a consequence of high *pr*_*bD*4_ values as it is evident by observing Fig. 7c produced by the same *in silico* experiment.

In order to investigate the independent influence of the two parameters in the system, a set of two additional parameter scans have been executed, keeping the other model parameters unchanged. In Fig. 7d, a set of 100 simulations are shown with different values for *pr*_*bD*4_, inside the range given in Table 3. The relationship between the parameter value and the G1/S Transition Time has been found to exhibit a progressively saturated ascending dynamic trend.

Regarding the relationship between *ModifierTime* and G1/S Transition Time or hypo-pRb detection time span clearly linear relationship can be observed in Figs. 7e and f as expected. These figures have been produced by a parameter scan (100 simulations) for *ModifierTime* parameter in the range [120, 1400] *min*. The aforementioned range has been produced bearing in mind the respective range of hypo-pRb positive cells (16 % ± 13.2 %) in an ALL patients’ population. This range starts from an almost complete disappearance and ends to a doubling in the number of cells compared to the mean case, for which the model has been calibrated with *ModifierTime* with a value equal to ~675 *min*. Once again, a uniform distribution of cells inside the cell cycle phases or sub-phases is assumed. It is remarked that, although the *ModifierTime* can significantly alter the time point at which the G1/S transition takes place, this parameter is not adequate to reproduce the observed variance of *T*_*c*_ in BCP-ALL, at least inside the range tested. Moreover, it should be mentioned that this range cannot be significantly widened, bearing in mind the percentage of hypo-pRb positive cells as explained above. In contrast, for the perturbation of *pr*_*bD*4_, a non-linear relationship between this parameter value and G1/S Transition Time exists. This results in a great variation of the model simulation endpoint, covering once again the estimated range for the G1 duration (Table 2). Therefore, although the combined perturbation of the parameters can explain the great variability of *T*_*c*_, this is primarily due to the influence of *pr*_*bD*4_ and the downstream effects of pseudo-hyper-pRb formation. As already mentioned, the phenomenon of Cdk4,6 substrate specificities deregulation (in which the pseudo-hyper-phosphorylation of pRb is based on), occurs among ALL patients to different extents. Hence, it can be inferred that the adoption of the proposed modifications of the system, not only allows the simulation of altered RP pathway dynamics, but also explains the increased variance of *T*_*c*_ in BCP-ALL.

The previous results imply that the model might be capable of predicting the cell cycle duration if some of its parameters, most probably the two ones tested above, are correlated with clinically or experimentally available data. This appears to be a particularly encouraging observation.

**Fig. 8:**
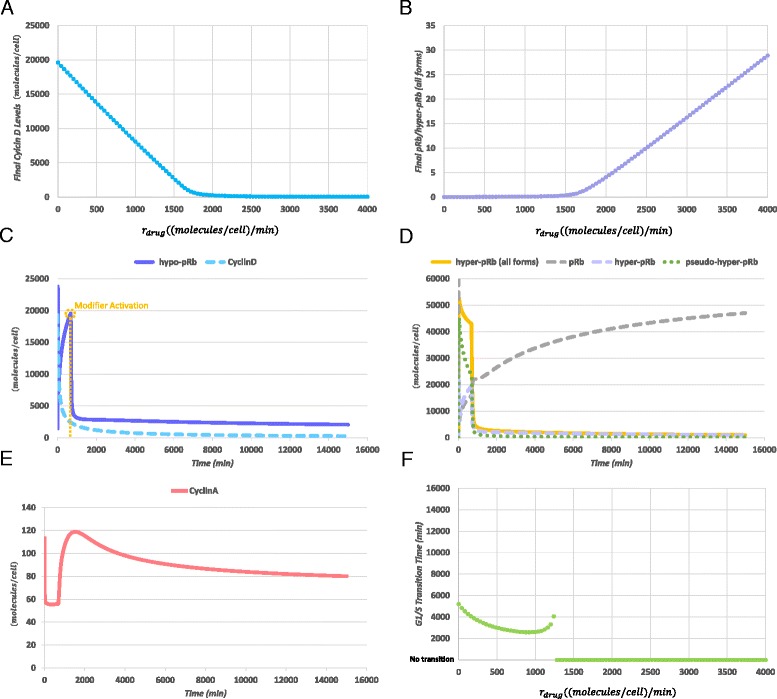
Parameter Scan and Simulation results for the drug administration scenario. (**a**) Relationship between Cyclin D levels (cyan) at the end of the simulation and drug administration rate (*r*_*drug*_ parameter value) (**b**) Relationship between the ratio of pRb vs. hyper-pRb (all forms) (dark purple) and *r*_*drug*_ parameter value. For an intermediate rate of drug administration: (**c**) free Cyclin D levels are rapidly diminished and hypo-pRb (purple) is predicted to show significant levels only for a limited period of time, (**d**) hyper-phosphorylated pRb species (all forms) (dark yellow) are rapidly de-phosphorylated leading to the increase, (**e**) Cyclin A levels fail to reach adequate for G1/S transition levels, (**f**) Relationship between G1/S Transition Time and *r*_*drug*_ parameter value. Small rates of drug administration leads to accelerated G1/S transition, however, after a certain threshold the cell cycle execution is slowed down and finally completely inhibited

The second scenario that has been implemented refers to the possible consequences of the administration of an anti-proliferative drug to the system dynamics. A widely used group of this type of drugs in ALL are the Glucocorticoids, mainly Prednisone and Dexamethasone, whose administration results in G1 cell cycle arrest and apoptotic death of leukemic cells [52]. It is noted that the potential relationship between these phenomena has not yet been fully elucidated. However, significant findings could be extracted from literature regarding the action mechanistic details of these drugs. Especially for the cell cycle arrest phenomenon, it has been shown that glucocorticoid drugs cytostatic properties are mediated by Cyclin D repression and retinoblastoma protein de-phosphorylation [67–71]. In order to render the model able to simulate such an intervention in the simplest possible way two new reactions and two new species have been introduced into the model presented in detail in Table 1 of Additional file 1 (reactions 139 and 140). The first reaction introduces the drug molecules into the system with a rate *r*_*drug*_ and the second one refers to the inhibition of free Cyclin D species by irreversible binding to the drug with a rate *r*_*drugBinding*_. Due to the lack of experimental data on one hand and the need to set the *r*_*drugBinding*_ parameter value within a realistic scale the value of the latter has been chosen to be similar to the rate at which Cdk4 binds to Cyclin D (*k*_*bCyclinDCdk*4_ in Table 1 of Additional file 1). Subsequently, a parameter scan procedure has been performed for the *r*_*drug*_ parameter in the range [0,4000 ((*molecules/cell*)/*min*). The values of the remaining model parameters remained unchanged. For each parameter scan step, the model has been simulated for 15000 *min* (250 *h*), in order to be able to identify any potential significantly delayed S-phase transition. The corresponding results are given in Fig. 8.

In Fig. 8a, the relationship between the Cyclin D levels at the end of the simulation and the *r*_*drug*_ parameter value is depicted. As can be observed, when *r*_*drug*_ approaches values greater than the half of the range tested, the free Cyclin D species are driven to very low or almost zero levels. A similar, though inversed, relationship with this parameter is also appointed for the ratio between un-phosphorylated pRb (pRb) and hyper-phosphorylated forms of pRb (hyper-pRb all forms) shown in Fig. 8b. From this figure it can be extracted that when free Cyclin D tends to reach values that render it almost fully inhibited, the un-phosphorylated form of retinoblastoma protein dominates the total levels of the protein. This is in agreement with what the literature dictates for these specific type of drugs administration results [70, 71]. An indicative simulation result of the model setting the *r*_*drug*_ parameter to 2000 ((*molecules/cell*)*/min*) is given in Fig. 8c, d and e. For this *in silico* experiment, it should be mentioned that the initiation of drug administration is assumed to be synchronized with the initiation of the cell cycle.

Finally, a result of exceptional interest is given in Fig. 8f. In this figure, the relationship between G1/S Transition Time and *r*_*drug*_ parameter, for the same parameter scan experiment, is shown. This relationship is found to exhibit a tri-phasic dynamic trend. In more detail, for values of *r*_*drug*_ in the range (0,1000] ((*molecules/cell*)*/min*), the G1/S Transition is predicted to happen earlier compared to the case where no drug is administered. Consequently, by further increasing the *r*_*drug*_ value, the G1/S Transition Time tends to recover until a sudden complete inhibition of cell cycle execution. This happens when the parameter reaches approximately the 1300 ((*molecules/cell*)*/min*) threshold. Taking into account the mechanistic details of the model, this behavior could be explained by a progressive shortage of available Cyclin D species in order for Cyclin D:Cdk4,6 complexes to be formulated, which in turn favor the creation of hypo-phosphorylated and pseudo-hyper-phosphorylated pRb species. These two pRb versions are able to delay the execution of the G1 phase, by inhibiting E2F transcription factors. However, an almost complete shortage in these species leads to an inability for hyper-phosphorylated forms of pRb to be created and a consequent cell cycle arrest. Interestingly, this *in silico* experimental finding is in agreement, at least in a qualitative context, with *in vitro* experimental findings where Prednisolone (the active metabolite of Prednisone) was found to exert mitogenic effects for low doses of the drug [72]. Although the aforementioned conclusion is not based on a mature analysis using experimental data and formulating a detailed model for the drug action, in our opinion, it definitely constitutes a first good indication of the soundness of the newly proposed model.

## Conclusions

In the framework of the present study the mechanistic details of the restriction point pathway, which predominantly regulate the execution of the G1-phase of the cell cycle and its possible deregulations in Acute Lymphoblastic Leukemia (focusing on BCP-ALL) have been thoroughly explored. An in depth review of the relevant literature has revealed the centrality of the almost complete domination of hyper-phosphorylated forms of retinoblastoma protein, compared to un-phosphorylated and hypo-phosphorylated versions. This appears to contradict the low proliferation rate of the leukemic cells which is lower than their normal counterparts, and the small percentage of lymphoblasts found in S-phase. A plausible hypothesis documented in literature suggests that this cell cycle related behavior could be the consequence of a partial inactivation of retinoblastoma protein functions, especially those concerning the differentiation program of the cell. Subsequently, based on this hypothesis, the hyper-phosphorylated forms of this biochemical entity may maintain their ability to inhibit the S-phase passage related transcription process. In that way, the leukemic cell population could become differentiation resistant while avoiding an excessive replication rate. The observed in ALL limited redundancy in retinoblastoma protein phosphorylation by the major Cyclin:Cdk complexes is believed to play a pivotal role in this restriction point reprogramming event.

In order to study *in silico* the aforementioned phenomena and hypotheses, specific modifications have been performed in an already published model for the biochemical regulation of the G1 phase of the cell cycle [40]. An additional objective has been to ensure the numerical agreement of the predicted time for the transition to S-phase with the one observed in ALL patients. A calibration procedure has been followed, in order to render the augmented model able to simulate the restriction point related deregulations in ALL and to be in numerical agreement with the reported mean cell kinetics and cell cycle dynamics in BCP-ALL. More specifically, a novel top-down and semi-quantitative/qualitative calibration procedure has been designed, incorporating global stochastic optimization methods, due to the lack of time course and quantitative protein level data. Moreover, after perturbing a set of its parameters, the model has been shown able to predict that the S-phase transition takes place within a realistic time range in agreement with the literature pertaining to the case of BCP-ALL. Finally, after *in silico* testing the interference in the cell cycle of a specific type of anti-leukemic drug (Glucocorticoids), the behavior of the system has been shown to be in line with the reported anti-proliferative consequences of the drug action.

These results provide a particularly good indication for the validity of the proposed model. A possible next step in the analysis will be a more precise calibration of the system using time course experimental data at the protein level sampled from leukemic cell populations. An additional future step will include the correlation of the model parameters with clinically available variables in order to enhance its capabilities in predicting personalized cell cycle duration values. This is crucial for the efficient parameterization of mechanistic multiscale cancer models, such as the (ALL) ISO&ISM_G Oncosimulator.

## Methods

### Reference model selection criteria

In order to select the cell cycle model that constituted the base for the development of the newly proposed model, specific criteria have been set. In Table 4 these criteria and their fulfillment by candidate models [40, 54–60, 73] are listed. Firstly, the detailed modeling of the restriction point machinery including the distinct pRb phosphorylation steps (in some models the hypo and hyper- phosphorylation steps are merged) has been set as a basic requirement. As presented in Background section, major deregulations of cell cycle in BCP-ALL are known to affect these mechanisms. Moreover, taking into account that the levels of Cyclin A were correlated with the percentage of leukemic cells entering the S-phase [32], models that simulate the expression of Cyclin A and its interference with the other cell cycle related biochemical entities, have been preferred. As far as the contribution of metabolism in cell cycle regulation is concerned, as discussed in Background, glycolysis rate is found to vary between two main groups of patients (good vs poor Prednisone responders) and to be correlated with cell growth and proliferation rate. Thus, a model that incorporates such a regulatory mechanism would allow the study of the influence of metabolism on the cell cycle dynamics of leukemic cells. Subsequently, the availability of the chosen model in a machine readable format (e.g. SBML), is thought to be crucial for the correct rebuilding of the model and the reproduction of its original results. In addition to the fulfillment of the above criteria, the creation of this model based on experiments where cells were exposed continuously to growth factors is thought to be closer to the *in vivo* setting.

**Table 4:** Reference model selection criteria

Model	Detailed pRb Phosphorylation steps modeling	Cyclin A levels modeling	Regulation of metabolism on the cell cycle execution modeling	Whole cell cycle machinery modeling	Referring to continuous growth factors exposure experiments	p16/p27 levels modeling	Availability in machine readable format
Yao et al. [60]	×	×	×	×	×	×/×	✓
Tyson and Novak [54] and Conradie et al. [55]	×	✓	✓	✓	×	×/✓	✓
Habericther et al. [40]	✓	✓	✓	×	✓	×/✓	✓
Swat et al. [59]	✓	×	×	×	×	×/×	✓
Iwamoto et al. (2008) [56]	✓	✓	×	×	-	✓/✓	×
Iwamoto et al. (2011) [57]	✓	✓	×	✓	-	✓/✓	×
Pfeuty [58]	✓	×	×	×	×	×/✓	×
Aguda and Tang [73]	×	×	×	×	-	✓/✓	✓

- : not defined

G1 phase is believed to be the most tunable phase of the cell cycle, as far as its duration is concerned [74]. As presented in Results & Discussion sections, there is concrete evidence that this holds true also for the case of BCP-ALL. Moreover, the major findings for the deregulations of cell cycle in ALL concern this phase. Therefore, although there are numerous models that describe the molecular regulation of the whole cell cycle machinery (not only of specific phases) this feature was not considered as absolutely necessary. Finally, p16 inactivation is significantly frequent in ALL (either by gene deletion or by methylation) [75]. Thus, regarding the modeling of p16 and p27 CdkI-related inhibitory phenomena on Cyclin D:Cdk4,6 and Cyclin E:Cdk2 complexes, a possible absence of this part of the pathway is not considered as important enough to affect the analysis performed in the present study, in contrast with p27.

Based on these criteria, the model chosen to be the base for the newly proposed model has been the one described in [40], since it fulfills the majority of the criteria set as significant.

## Model implementation and simulation

The reference model was acquired by accessing the BIOMD0000000109 entry in BIOMODELS database [76] and was verified using the relevant Ordinary Differential Equations provided in [40] by the SBML compatible modeling and simulation tool COPASI [63] (Versions 4.12, 4.13 and 4.14). The additions in order for the newly proposed model to be derived have been also done in COPASI. The SBML version of the model was created using the same tool (given in Additional file 2). Finally the reference and the newly proposed models have been simulated by the same tool using the Deterministic (LSODA) simulation algorithm, choosing an interval size of 10 *min*.

### Graphs and figures creation

The illustrations shown in Figs. 1, 2 and 5 were created using Microsoft Visio 2013. Additionally, the graph in Fig. 4 was produced using the SBGN capabilities of Cell Designer tool [77, 78] (Versions 4.3 and 4.4). Finally the graphical representations of model simulation results in Figs. 3, 6, 7 and 8 were produced by Microsoft Excel 2013.

### Semi-quantitative/qualitative criteria, objective function definition, calibration method and results

In order for the semi-quantitative/qualitative criteria to be evaluated, the Events system in COPASI has been used. More specifically, four distinct events that, by monitoring the Time of the simulation, change the values of seven distinct flags have been introduced. These flags refer to the distinct time intervals as given in Fig. 5. Finally, their values for every time interval are given in Table 5.

**Table 5:** Definition of calibration flags

Time Interval	Flags						
	*Pre*_*Modifier*_	*Post*_*Modifier*_	*Flag*_*0*_	*Flag*_*1*_	*Flag*_*2*_	*Flag*_*3*_	*Flag*_*4*_
(*Time ≥ 0*) & (*Time < ModifierTime*)	1	0	1	0	0	0	0
(*Time ≥ ModifierTime*) & (*Time < hypo-pRb detection time span*)	0	1	0	1	0	0	0
(*Time ≥ hypo-pRb detection time span*) & (*Time < G1/S Transition Time Objective*)	0	1	0	0	1	0	0
(*Time ≥ G1/S Transition Time Objective*) & (*Time < S-phase-midpoint*)	0	1	0	0	0	1	0
*Time ≥ S-phase-midpoint*	0	1	0	0	0	0	1

Using the flags defined above, the penalties given in Tables 6 and 7 could be calculated for the species involved in the semi-quantitative/qualitative criteria.

**Table 6:** Definition of calibration penalties related to Cyclin A and Cyclin D levels

Cyclin A Levels	
$$ \begin{array}{l}Cyc{A}_{penalt{y}_i}={\displaystyle \sum_{Time=0}^{Time= SimulationTime}Cyc{A}_{instantpenalt{y}_i}}\\ {}\kern9.5em i=0,1,2,3,4\kern3em \end{array} $$	$$ Cyc{A}_{instant\ penalt{y}_0}=\left\{\begin{array}{c}\hfill CyclinA>150\ \left( molecules/ cell\right),\ Fla{g}_0\hfill \\ {}\hfill 0\hfill \end{array}\right. $$
	$$ Cyc{A}_{instant\ penalt{y}_1}=\left\{\begin{array}{c}\hfill CyclinA>300\ \left( molecules/ cell\right),\ Fla{g}_1\hfill \\ {}\hfill 0\hfill \end{array}\right. $$
	$$ Cyc{A}_{instant\ penalt{y}_2}=\left\{\begin{array}{c}\hfill CyclinA>300\ \left( molecules/ cell\right),\ Fla{g}_2\hfill \\ {}\hfill 0\hfill \end{array}\right. $$
	$$ Cyc{A}_{instant\ penalt{y}_3}=\left\{\begin{array}{c}\hfill CyclinA<300\ \left( molecules/ cell\right),\ Fla{g}_3\hfill \\ {}\hfill 0\hfill \end{array}\right. $$
	$$ Cyc{A}_{instant\ penalt{y}_4}=\left\{\begin{array}{c}\hfill CyclinA<300\ \left( molecules/ cell\right),\ Fla{g}_4\hfill \\ {}\hfill 0\hfill \end{array}\right. $$
$$ Cyc{A}_{penalt{y}_{sum}}={\displaystyle \sum_i}Cyc{A_{penalt y}}_{{}_i},\ i=0,1,2,3,4 $$	
Cyclin A Rate	
$CycA\ Rat{e}_{penalty}={\displaystyle \sum_{Time=0}^{Time= Simulation\ Time}} CycA\ Rat{e}_{instant\ penalt{y}_0}$	$$ CycA\ Rat{e}_{instant\ penalt{y}_0}=\left\{\begin{array}{c}\hfill \frac{d\ CyclinA}{dt}>0\ \left(\left( molecules/ cell\right)/ min\right),\ Fla{g}_0\hfill \\ {}\hfill 0\hfill \end{array}\right. $$
Cyclin D Levels	
$CycD\ Va{r}_{penalty}={\displaystyle \sum_{Time=0}^{Time= Simulation\ Time}} CycD\ Va{r}_{instant\ penalty}$	$$ CycD\ Va{r}_{instant\ penalty}=\left\{\begin{array}{c}\hfill Cyc{D}_{initial\ levels}-Cyc{D}_{instant\ levels}>500\left( molecules/ cell\right),\ 1\hfill \\ {}\hfill 0\hfill \end{array}\right. $$

**Table 7:** Definition of calibration penalties related to pRb Levels

hypo-pRb Levels	
$$ \begin{array}{l} hypo-pR{b}_{penalt{y}_i}={{\displaystyle \sum_{Time=0}^{Time= SimulationTime} hypo-pRb}}_{instantpenalt{y}_i}\\ {}\kern13em i=0,1,2,3,4\end{array} $$	$$ hypo-pR{b}_{instant\ penalt{y}_0}=\left\{\begin{array}{c}\hfill hypo-pRb< hypo-pRb\ detection\ levels,\kern0.5em Fla{g}_0\hfill \\ {}\hfill 0\hfill \end{array}\right. $$
	$$ hypo-pR{b}_{instant\ penalt{y}_1}=\left\{\begin{array}{c}\hfill hypo-pRb\ge hypo-pRb\ detection\ levels,\kern0.5em Fla{g}_1\hfill \\ {}\hfill 0\hfill \end{array}\right. $$
	$$ hypo-pR{b}_{instant\ penalt{y}_2}=\left\{\begin{array}{c}\hfill hypo-pRb\ge hypo-pRb\ detection\ levels,\kern0.5em Fla{g}_2\hfill \\ {}\hfill 0\hfill \end{array}\right. $$
	$$ hypo-pR{b}_{instant\ penalt{y}_3}=\left\{\begin{array}{c}\hfill hypo-pRb\ge hypo-pRb\ detection\ levels,\kern0.5em Fla{g}_3\hfill \\ {}\hfill 0\hfill \end{array}\right. $$
	$$ hypo-pR{b}_{instant\ penalt{y}_4}=\left\{\begin{array}{c}\hfill hypo-pRb\ge hypo-pRb\ detection\ levels,\kern0.5em Fla{g}_4\hfill \\ {}\hfill 0\hfill \end{array}\right. $$
$$ hypo-pR{b}_{penalt{y}_{sum}}={\displaystyle \sum_i} hypo-pR{b}_{penalt{y}_i},\ i=0,1,2,3,4 $$	
(pRb + hypo-pRb) vs. hyper-pRb (all forms) levels	
$$ \begin{array}{l}\left(pRb+ hypo-pRb\right)\;vs.\; hyper-pR{b}_{penalt{y}_i}={\displaystyle \sum_{Time=0}^{Time= SimulationTime}\left(pRb+ hypo-pRb\right)}\;vs.\; hyper-pR{b}_{instantpenalt{y}_i}\\ {}\kern32.5em i=0,1\end{array} $$	$$ \left(pRb+ hypo-pRb\right)vs.\ hyper-pR{b}_{instant\ penalt{y}_0}=\left\{\begin{array}{c}\hfill hyper-pRb\ \left( all\ forms\right)>pRb+ hypo-pRb, \kern0.5em 0\hfill \\ {}\hfill Pre\_ Modifier\hfill \end{array}\right. $$
	$$ \left(pRb+ hypo-pRb\right)vs.\ hyper-pR{b}_{instant\ penalt{y}_1}=\left\{\begin{array}{c}\hfill hyper-pRb\ \left( all\ forms\right)>pRb+ hypo-pRb, \kern0.5em 0\hfill \\ {}\hfill Post\_ Modifier\hfill \end{array}\right. $$

Having defined the above penalties, the following objective function has been formulated in order to be minimized:

$$ Objective\  Function\ \left({\boldsymbol{x}}_{\boldsymbol{j}}\right) = Cyc{A}_{penalt{y}_{sum}}\left({\boldsymbol{x}}_{\boldsymbol{j}}\right)+ CycA\  Rat{e}_{penalt y}\left({\boldsymbol{x}}_{\boldsymbol{j}}\right)+ hypo-pR{b}_{penalt{y}_{sum}}\left({\boldsymbol{x}}_{\boldsymbol{j}}\right) + CycD\ Va{r}_{penalt y}\ \left({\boldsymbol{x}}_{\boldsymbol{j}}\right) $$

Constrained by:

$$ 0\le \left(pRb+ hypo-pRb\right)vs.\  hyper-pR{b}_{penalt{y}_0}\le 300 $$

$$ 0\le \left(pRb+ hypo-pRb\right)vs.\  hyper-pR{b}_{penalt{y}_1}\le 100 $$

where *x*_*j*_ is a specific parameterization of the model.

A precise measurement of the form of pRb protein that prevails in the various G1 sub-phases was not found in literature. Therefore, the upper bounds in the above defined constraints were empirically chosen in order to represent our estimation of the maximum time points for which hyper-phosphorylated pRb levels are outweighed by the levels of the un- and hypo-phosphorylated forms.

The objective function has been introduced into COPASI optimization task, and the Particle Swarm global optimization algorithm, as provided in the tool and with the default parameters, has been used for its minimization. This was realized by perturbing a set of model parameters as described in Calibration of the model sub-section of Results and discussion. The initial values of the parameters have been randomly chosen by the tool, within their range in the parameter space. By executing the optimization procedure, the algorithm concluded to a value for the objective function equal to 28.07651751 and the results of this procedure are given in Table 3 of the same sub-section.

### Availability of supporting data

The semi-quantitative and the qualitative data supporting the results of this article are included and cited within the article and its additional files. The model used as the base for the development of the proposed model (reference model) is taken from [40] and can be retrieved from the BioModels Database at https://www.ebi.ac.uk/biomodels-main/ where it is stored as BIOMD0000000109. The model developed in this study is available as a supplemental SBML file (Additional file 3).

## Additional files

Additional file 1: Table S1. List of model reactions and kinetic parameters. **Table S2.** Initial levels of the model species. (PDF 477 kb) Additional file 2:
**Detailed description of the model calibration criteria derivation process.** (PDF 458 kb) Additional file 3:
**SBML description of the model.** (XML 573 kb)

## Abbreviations

ALL: acute lymphoblastic leukemia
BCP-ALL: precursor-B acute lymphoblastic leukemia
Cdk: cyclin dependent kinase
CdkI: cyclin dependent kinase inhibitor
COPASI: COmplex PAthway Simulator
hyper-pRb: hyper-phosphorylated pRb
hypo-pRb: hypo-phosphorylated pRb
ISO&ISM_G: in silico oncology and in silico medicine group
pRb: retinoblastoma protein (unphosphorylated)
pseudo-hyper-pRb: pseudo-hyperphosphorylated pRb
RP: restriction point
