# Supplementary material for: Differentiation resistance through altered retinoblastoma protein function in acute lymphoblastic leukemia: in silico modeling of the deregulations in the G1/S restriction point pathway
Source: BMC Syst Biol. 2016 Mar 1;10:23. doi: 10.1186/s12918-016-0264-5 (PMC4774111; doi:10.1186/s12918-016-0264-5)
Supplement: Additional file 1: Table S1. — List of model reactions and kinetic parameters. Table S2. Initial levels of the model species. (PDF 477 kb) [file 12918_2016_264_MOESM1_ESM.pdf]

Additional file 1

Differentiation resistance through altered retinoblastoma protein function in Acute Lymphoblastic Leukemia: In silico modeling of the deregulations in the G1/S restriction point pathway

Eleftherios Ouzounoglou, Dimitra Dionysiou and Georgios S. Stamatakos

Table S1 List of model reactions and kinetic parameters

Reaction Number	Reaction	Rate Law	Parameter Name and Value or Definition
1	-> CyclinD	Constant Flux	$k_{sCyclinD} = 1761.08\ ((molecules/cell)/min)$
2	CyclinD ->	Mass Action	$k_{dCyclinD} = 0.05\ (min)^{-1}$
3	Cyclin D:Cdk 4 -> Cdk 4	Mass Action	$k_{dCyclinD} = 0.05\ (min)^{-1}$
4	p27:Cyclin D:Cdk 4 -> p27:Cdk 4	Mass Action	$k_{dCyclinD} = 0.05\ (min)^{-1}$
5	-> p27	Constant Flux	$k_{sp27} = 195.472\ ((molecules/cell)/min)$
6	p27 ->	Mass Action	$k_{dp27} = 0.001575\ (min)^{-1}$
7	p27:Cdk 4 -> Cdk 4	Mass Action	$k_{dp27} = 0.001575\ (min)^{-1}$
8	p27:Cyclin D:Cdk 4 -> Cyclin D:Cdk 4	Mass Action	$k_{dp27} = 0.001575\ (min)^{-1}$
9	p27:Cdk2 -> Cdk 2	Mass Action	$k_{dp27} = 0.001575\ (min)^{-1}$
10	p27:Cyclin E:Cdk2 -> Cyclin E:Cdk2	Mass Action	$k_{dp27} = 0.001575\ (min)^{-1}$
11	p27:Cyclin A:Cdk2 -> Cyclin A:Cdk2	Mass Action	$k_{dp27} = 0.001575\ (min)^{-1}$
12	p27:Cdk2(M) -> Cdk2(M)	Mass Action	$k_{dp27} = 0.001575\ (min)^{-1}$
13	p27:Cyclin E:Cdk2(M) -> Cyclin E:Cdk2(M)	Mass Action	$k_{d1p27} = 0.071149\ (min)^{-1}$
14	p27:Cyclin A:Cdk2(M) -> Cyclin A:Cdk2(M)	Mass Action	$k_{dp27} = 0.001575\ (min)^{-1}$
15	-> CyclinE	Constant Flux	$k_{sCyclinE} = k_{s0CyclinE} + \frac{k_{s1CyclinE} * [E2F]}{k_{sMCyclinE} + [E2F]}$<br>$k_{s0CyclinE} = 254.0742$<br>$k_{s1CyclinE} = 980.611$<br>$k_{sMCyclinE} = 9992.647$
16	CyclinE ->	Mass Action	$k_{dCyclinE} = 0.05\ (min)^{-1}$
17	Cyclin E:Cdk2 -> Cdk 2	Mass Action	$k_{dCyclinE} = 0.05\ (min)^{-1}$
18	p27:Cyclin E:Cdk2 -> p27:Cdk2	Mass Action	$k_{dCyclinE} = 0.05\ (min)^{-1}$
19	Cyclin E:Cdk2(M) -> Cdk2(M)	Mass Action	$k_{dCyclinE} = 0.05\ (min)^{-1}$
20	p27:Cyclin E:Cdk2(M) -> p27:Cdk2(M)	Mass Action	$k_{dCyclinE} = 0.05\ (min)^{-1}$
21	-> CyclinA	Constant Flux	$k_{sCyclinA} = k_{s0CyclinA} + \frac{k_{s1CyclinA} * [E2F]}{k_{sMCyclinA} + [E2F]}$<br>$k_{s0CyclinA} = 499.9437$<br>$k_{s1CyclinA} = 7999.996$<br>$k_{sMCyclinA} = 4064.384$
22	CyclinA ->	Mass Action	$k_{dCyclinA} = 0.05\ (min)^{-1}$
23	Cyclin A:Cdk1 -> Cdk1	Mass Action	$k_{dCyclinA} = 0.05\ (min)^{-1}$
24	Cyclin A:Cdk1(M) -> Cdk1(M)	Mass Action	$k_{dCyclinA} = 0.05\ (min)^{-1}$
25	Cyclin A:Cdk2 -> Cdk 2	Mass Action	$k_{dCyclinA} = 0.05\ (min)^{-1}$
26	p27:Cyclin A:Cdk2 -> p27:Cdk2	Mass Action	$k_{dCyclinA} = 0.05\ (min)^{-1}$
27	Cyclin A:Cdk2(M) -> Cdk2(M)	Mass Action	$k_{dCyclinA} = 0.05\ (min)^{-1}$
28	p27:Cyclin A:Cdk2(M) -> p27:Cdk2(M)	Mass Action	$k_{dCyclinA} = 0.05\ (min)^{-1}$
29	-> E2F	Constant Flux	$k_{sE2F} = k_{s0E2F} + \frac{k_{s1E2F} * [E2F]}{k_{sME2F} + [E2F]}$<br>$k_{s0E2F} = 6.927086$<br>$k_{s1CyclinA} = 65.44282$<br>$k_{smCyclinA} = 9818.78$

30	E2F ->	Mass Action	$k_{d0E2F} = 0.002229\ (min)^{-1}$
31	E2F:pRb -> pRb	Mass Action	$k_{dE2F} = 0.006465\ (min)^{-1}$
32	E2F:hypo-pRb -> hypo-pRb	Mass Action	$k_{dE2F} = 0.006465\ (min)^{-1}$
33	E2F:hyper-pRb -> hyper-pRb	Mass Action	$k_{dE2F} = 0.006465\ (min)^{-1}$
33_leuk	E2F:pseudo-hyper-pRb -> pseudo-hyper-pRb	Mass Action	$k_{dE2F_{leuk}} = pr_{dE2F} * k_{dE2F}$<br>$pr_{dE2F} = 1$
34	-> Emi1	Constant Flux	$k_{sEmi1} = k_{s0Emi1} + \frac{k_{s1Emi1} * [E2F]}{k_{sMEmi1} + [E2F]}$<br>$k_{s0Emi1} = 2.004744$<br>$k_{s1Emi1} = 1788.517$<br>$k_{sMEmi1} = 9608.162$
35	Emi1 ->	Mass Action	$k_{dEmi1} = 0.018158\ (min)^{-1}$
36	APCC:Emi1 -> APCC	Mass Action	$k_{dEmi1} = 0.018158\ (min)^{-1}$
37	Cdk 4 + CyclinD -> Cyclin D:Cdk 4	Mass Action	$k_{bCyclinDCdk4} = 1.43 \cdot e^{-6} \left(\left(\frac{molecules}{cell}\right) * min\right)^{-1}$
38	Cyclin D:Cdk 4 -> Cdk 4 + CyclinD	Mass Action	$k_{uCyclinDCdk4} = 0.1\ (min)^{-1}$
39	p27:Cdk 4 + CyclinD -> p27:Cyclin D:Cdk 4	Mass Action	$k_{bCyclinDCdk4} = 1.43 \cdot e^{-6} \left(\left(\frac{molecules}{cell}\right) * min\right)^{-1}$
40	p27:Cyclin D:Cdk 4 -> p27:Cdk 4 + CyclinD	Mass Action	$k_{uCyclinDCdk4} = 0.1\ (min)^{-1}$
41	Cdk 4 + p27 -> p27:Cdk 4	Mass Action	$k_{bp27Cdk4} = 6.34 \cdot e^{-6} \left(\left(\frac{molecules}{cell}\right) * min\right)^{-1}$
42	p27:Cdk 4 -> Cdk 4 + p27	Mass Action	$k_{up27Cdk4} = 0.1\ (min)^{-1}$
43	Cyclin D:Cdk 4 + p27 -> p27:Cyclin D:Cdk 4	Mass Action	$k_{bp27Cdk4} = 6.34 \cdot e^{-6} \left(\left(\frac{molecules}{cell}\right) * min\right)^{-1}$
44	p27:Cyclin D:Cdk 4 -> Cyclin D:Cdk 4 + p27	Mass Action	$k_{bp27Cdk4} = 6.34 \cdot e^{-6} \left(\left(\frac{molecules}{cell}\right) * min\right)^{-1}$
45	Cdk 2 + p27 -> p27:Cdk2	Mass Action	$k_{bp27Cdk2} = 1.23 \cdot e^{-5} \left(\left(\frac{molecules}{cell}\right) * min\right)^{-1}$
46	p27:Cdk2 -> Cdk 2 + p27	Mass Action	$k_{up27Cdk2} = 0.1\ (min)^{-1}$
47	Cyclin E:Cdk2 + p27 -> p27:Cyclin E:Cdk2	Mass Action	$k_{bp27Cdk2} = 1.23 \cdot e^{-5} \left(\left(\frac{molecules}{cell}\right) * min\right)^{-1}$
48	p27:Cyclin E:Cdk2 -> Cyclin E:Cdk2 + p27	Mass Action	$k_{up27Cdk2} = 0.1\ (min)^{-1}$
49	Cyclin A:Cdk2 + p27 -> p27:Cyclin A:Cdk2	Mass Action	$k_{bp27Cdk2} = 1.23 \cdot e^{-5} \left(\left(\frac{molecules}{cell}\right) * min\right)^{-1}$
50	p27:Cyclin A:Cdk2 -> Cyclin A:Cdk2 + p27	Mass Action	$k_{up27Cdk2} = 0.1\ (min)^{-1}$
51	Cdk2(M) + p27 -> p27:Cdk2(M)	Mass Action	$k_{bp27Cdk2} = 1.23 \cdot e^{-5} \left(\left(\frac{molecules}{cell}\right) * min\right)^{-1}$
52	p27:Cdk2(M) -> Cdk2(M) + p27	Mass Action	$k_{up27Cdk2} = 0.1\ (min)^{-1}$
53	Cyclin E:Cdk2(M) + p27 -> p27:Cyclin E:Cdk2(M)	Mass Action	$k_{bp27Cdk2} = 1.23 \cdot e^{-5} \left(\left(\frac{molecules}{cell}\right) * min\right)^{-1}$
54	p27:Cyclin E:Cdk2(M) -> Cyclin E:Cdk2(M) + p27	Mass Action	$k_{up27Cdk2} = 0.1\ (min)^{-1}$
55	Cyclin A:Cdk2(M) + p27 -> p27:Cyclin A:Cdk2(M)	Mass Action	$k_{bp27Cdk2} = 1.23 \cdot e^{-5} \left(\left(\frac{molecules}{cell}\right) * min\right)^{-1}$
56	p27:Cyclin A:Cdk2(M) -> Cyclin A:Cdk2(M) + p27	Mass Action	$k_{up27Cdk2} = 0.1\ (min)^{-1}$
57	Cdk 2 -> Cdk2(M)	Mass Action	$k_{act} = \begin{cases} Time < ModifierTime, 0 \\ Time \geq ModifierTime, 0.0175 \end{cases} (min)^{-1}$
58	p27:Cdk2 -> p27:Cdk2(M)	Mass Action	$k_{act} = \begin{cases} Time < ModifierTime, 0 \\ Time \geq ModifierTime, 0.0175 \end{cases} (min)^{-1}$
59	Cyclin E:Cdk2 -> Cyclin E:Cdk2(M)	Mass Action	$k_{act} = \begin{cases} Time < ModifierTime, 0 \\ Time \geq ModifierTime, 0.0175 \end{cases} (min)^{-1}$
60	p27:Cyclin E:Cdk2 -> p27:Cyclin E:Cdk2(M)	Mass Action	$k_{act} = \begin{cases} Time < ModifierTime, 0 \\ Time \geq ModifierTime, 0.0175 \end{cases} (min)^{-1}$
61	Cyclin A:Cdk2 -> Cyclin A:Cdk2(M)	Mass Action	$k_{act} = \begin{cases} Time < ModifierTime, 0 \\ Time \geq ModifierTime, 0.0175 \end{cases} (min)^{-1}$
62	p27:Cyclin A:Cdk2 -> p27:Cyclin A:Cdk2(M)	Mass Action	$k_{act} = \begin{cases} Time < ModifierTime, 0 \\ Time \geq ModifierTime, 0.0175 \end{cases} (min)^{-1}$
63	Cdk 2 + CyclinE -> Cyclin E:Cdk2	Mass Action	$k_{bCyclinECdk2} = 5.01 \cdot e^{-5} \left(\left(\frac{molecules}{cell}\right) * min\right)^{-1}$
64	Cyclin E:Cdk2 -> Cdk 2 + CyclinE	Mass Action	$k_{uCyclinECdk2} = 0.1\ (min)^{-1}$
65	p27:Cdk2 + CyclinE -> p27:Cyclin E:Cdk2	Mass Action	$k_{bCyclinECdk2} = 5.01 \cdot e^{-5} \left(\left(\frac{molecules}{cell}\right) * min\right)^{-1}$
66	p27:Cyclin E:Cdk2 -> p27:Cdk2 + CyclinE	Mass Action	$k_{uCyclinECdk2} = 0.1\ (min)^{-1}$
67	Cdk2(M) + CyclinE -> Cyclin E:Cdk2(M)	Mass Action	$k_{bCyclinECdk2} = 5.01 \cdot e^{-5} \left(\left(\frac{molecules}{cell}\right) * min\right)^{-1}$

68	Cyclin E:Cdk2(M) -> Cdk2(M) + CyclinE	Mass Action	$k_{uCyclinECdk2} = 0.1\ (min)^{-1}$
69	p27:Cdk2(M) + CyclinE -> p27:Cyclin E:Cdk2(M)	Mass Action	$k_{bCyclinECdk2} = 5.01 \cdot e^{-5} \left(\left(\frac{molecules}{cell}\right) * min\right)^{-1}$
70	p27:Cyclin E:Cdk2(M) -> p27:Cdk2(M) + CyclinE	Mass Action	$k_{uCyclinECdk2} = 0.1\ (min)^{-1}$
71	Cdk 2 + CyclinA -> Cyclin A:Cdk2	Mass Action	$k_{bCyclinACdk2} = 9.52 \cdot e^{-5} \left(\left(\frac{molecules}{cell}\right) * min\right)^{-1}$
72	Cyclin A:Cdk2 -> Cdk 2 + CyclinA	Mass Action	$k_{uCyclinACdk2} = 0.1\ (min)^{-1}$
73	p27:Cdk2 + CyclinA -> p27:Cyclin A:Cdk2	Mass Action	$k_{bCyclinACdk2} = 9.52 \cdot e^{-5} \left(\left(\frac{molecules}{cell}\right) * min\right)^{-1}$
74	p27:Cyclin A:Cdk2 -> p27:Cdk2 + CyclinA	Mass Action	$k_{uCyclinACdk2} = 0.1\ (min)^{-1}$
75	Cdk2(M) + CyclinA -> Cyclin A:Cdk2(M)	Mass Action	$k_{bCyclinACdk2} = 9.52 \cdot e^{-5} \left(\left(\frac{molecules}{cell}\right) * min\right)^{-1}$
76	Cyclin A:Cdk2(M) -> Cdk2(M) + CyclinA	Mass Action	$k_{uCyclinACdk2} = 0.1\ (min)^{-1}$
77	p27:Cdk2(M) + CyclinA -> p27:Cyclin A:Cdk2(M)	Mass Action	$k_{bCyclinACdk2} = 9.52 \cdot e^{-5} \left(\left(\frac{molecules}{cell}\right) * min\right)^{-1}$
78	p27:Cyclin A:Cdk2(M) -> p27:Cdk2(M) + CyclinA	Mass Action	$k_{uCyclinACdk2} = 0.1\ (min)^{-1}$
79	Cdk1 -> Cdk1(M)	Mass Action	$k_{act} = \begin{cases} Time < ModifierTime, 0 \\ Time \geq ModifierTime, 0.0175 \end{cases} (min)^{-1}$
80	Cyclin A:Cdk1 -> Cyclin A:Cdk1(M)	Mass Action	$k_{act} = \begin{cases} Time < ModifierTime, 0 \\ Time \geq ModifierTime, 0.0175 \end{cases} (min)^{-1}$
81	Cdk1 + CyclinA -> Cyclin A:Cdk1	Mass Action	$k_{bCyclinACdk1} = 6.48 \cdot e^{-5} \left(\left(\frac{molecules}{cell}\right) * min\right)^{-1}$
82	Cyclin A:Cdk1 -> Cdk1 + CyclinA	Mass Action	$k_{uCyclinACdk1} = 0.1\ (min)^{-1}$
83	Cdk1(M) + CyclinA -> Cyclin A:Cdk1(M)	Mass Action	$k_{bCyclinACdk1} = 6.48 \cdot e^{-5} \left(\left(\frac{molecules}{cell}\right) * min\right)^{-1}$
84	Cyclin A:Cdk1(M) -> Cdk1(M) + CyclinA	Mass Action	$k_{uCyclinACdk1} = 0.1\ (min)^{-1}$
85	pRb + Cyclin D:Cdk 4 -> Cyclin D:Cdk 4_pRb_hypo-pRb_Int	Mass Action	$k_{bD4pRb} = 3.1 \cdot e^{-5} \left(\left(\frac{molecules}{cell}\right) * min\right)^{-1}$
85_leuk_1	E2F:hypo-pRb + Cyclin D:Cdk 4 -> Cyclin D:Cdk 4_E2F:hypo-pRb_E2F:pseudo-hyper-pRb_Int	Mass Action	${k_{bD4pRb}}_{leuk} = pr_{bD4} * k_{bD4pRb}$<br>$pr_{bD4} = 0.8416992397$
85_leuk_2	hypo-pRb + Cyclin D:Cdk 4 -> Cyclin D:Cdk 4_hypo-pRb_pseudo-hyper-pRb_Int	Mass Action	${k_{bD4pRb}}_{leuk} = pr_{bD4} * k_{bD4pRb}$<br>$pr_{bD4} = 0.8416992397$
86	Cyclin D:Cdk 4_pRb_hypo-pRb_Int -> pRb + Cyclin D:Cdk 4	Mass Action	$k_{uD4pRb} = 0.1\ (min)^{-1}$
86_leuk_1	Cyclin D:Cdk 4_E2F:hypo-pRb_E2F:pseudo-hyper-pRb_Int -> E2F:hypo-pRb + Cyclin D:Cdk 4	Mass Action	${k_{uD4pRb}}_{leuk} = pr_{uD4} * k_{uD4pRb}$<br>$pr_{uD4} = 1$
86_leuk_2	Cyclin D:Cdk 4_hypo-pRb_pseudo-hyper-pRb_Int -> hypo-pRb + Cyclin D:Cdk 4	Mass Action	${k_{uD4pRb}}_{leuk} = pr_{uD4} * k_{uD4pRb}$<br>$pr_{uD4} = 1$
87	Cyclin D:Cdk 4_pRb_hypo-pRb_Int -> hypo-pRb + Cyclin D:Cdk 4	Mass Action	$k_{upD4pRb} = 1.69466\ (min)^{-1}$
87_leuk_1	Cyclin D:Cdk 4_ E2F:hypo-pRb_ E2F:pseudo-hyper-pRb_Int -> E2F:pseudo-hyper-pRb + Cyclin D:Cdk 4	Mass Action	${k_{upD4pRb}}_{leuk} = pr_{upD4} * k_{upD4pRb}$<br>$pr_{upD4} = 0.484117838$
87_leuk_2	Cyclin D:Cdk 4_hypo-pRb_pseudo-hyper-pRb_Int -> pseudo-hyper-pRb + Cyclin D:Cdk 4	Mass Action	${k_{uD4pRb}}_{leuk} = pr_{uD4} * k_{uD4pRb}$<br>$pr_{uD4} = 1$
88	E2F:pRb + Cyclin D:Cdk 4 -> Cyclin D:Cdk 4_E2F:pRb_E2F:hypo-pRb_Int	Mass Action	$k_{bD4pRb} = 3.1 \cdot e^{-5} \left(\left(\frac{molecules}{cell}\right) * min\right)^{-1}$
89	Cyclin D:Cdk 4_E2F:pRb_E2F:hypo-pRb_Int -> E2F:pRb + Cyclin D:Cdk 4	Mass Action	$k_{uD4pRb} = 0.1\ (min)^{-1}$
90	Cyclin D:Cdk 4_E2F:pRb_E2F:hypo-pRb_Int -> E2F:hypo-pRb + Cyclin D:Cdk 4	Mass Action	$k_{upD4pRb} = 1.69466\ (min)^{-1}$
91	hypo-pRb + Cyclin E:Cdk2(M) -> CyclinE:Cdk2(M)_hypo-pRb_hyper-pRb_Int	Mass Action	$k_{bE2pRb} = 5.74 \cdot e^{-5} \left(\left(\frac{molecules}{cell}\right) * min\right)^{-1}$
91_leuk	pseudo-hyper-pRb + Cyclin E:Cdk2(M) -> CyclinE:Cdk2(M)_pseudo-hyper-pRb_hyper-pRb_Int	Mass Action	$k_{bE2pRb_{leuk}} = pr_{bE2A2A1} * k_{bE2pRb}$<br>$pr_{bE2A2A1} = 0.1301449788$
92	Cyclin E:Cdk2(M)_hypo-pRb_hyper-pRb_Int -> hypo-pRb + Cyclin E:Cdk2(M)	Mass Action	$k_{uE2pRb} = 0.1\ (min)^{-1}$
92_leuk	Cyclin E:Cdk2(M)_pseudo-hyper-pRb_hyper-pRb_Int -> pseudo-hyper-pRb + Cyclin E:Cdk2(M)	Mass Action	$k_{uE2pRb_{leuk}} = pr_{uE2A2A1} * k_{uE2pRb}$<br>$pr_{uE2A2A1} = 1$
93	Cyclin E:Cdk2(M)_hypo-pRb_hyper-pRb_Int -> hyper-pRb + Cyclin E:Cdk2(M)	Mass Action	$k_{upE2pRb} = 4.78271\ (min)^{-1}$
93_leuk	Cyclin E:Cdk2(M)_pseudo-hyper-pRb_hyper-pRb_Int -> hyper-pRb + Cyclin E:Cdk2(M)	Mass Action	$k_{upE2pRb_{leuk}} = pr_{upE2A2A1} * k_{upE2pRb}$<br>$pr_{upE2A2A1} = 0.428204395$
94	E2F:hypo-pRb + Cyclin E:Cdk2(M) -> Cyclin E:Cdk2(M)_E2F:hypo-pRb_E2F:hyper-pRb_Int	Mass Action	$k_{bE2pRb} = 5.74 \cdot e^{-5} \left(\left(\frac{molecules}{cell}\right) * min\right)^{-1}$
94_leuk	E2F:pseudo-hyper-pRb + Cyclin E:Cdk2(M) -> Cyclin E:Cdk2(M)_E2F:pseudo-hyper-pRb_E2F:hyper-pRb_Int	Mass Action	$k_{bE2pRb_{leuk}} = pr_{bE2A2A1} * k_{bE2pRb}$<br>$pr_{bE2A2A1} = 0.1301449788$
95	Cyclin E:Cdk2(M)_E2F:hypo-pRb_E2F:hyper-pRb_Int -> E2F:hypo-pRb + Cyclin E:Cdk2(M)	Mass Action	$k_{uE2pRb} = 0.1\ (min)^{-1}$
95_leuk	Cyclin E:Cdk2(M)_E2F:pseudo-hyper-pRb_E2F:hyper-pRb_Int -> E2F:pseudo-hyper-pRb + Cyclin E:Cdk2(M)	Mass Action	$k_{uE2pRb_{leuk}} = pr_{uE2A2A1} * k_{uE2pRb}$<br>$pr_{uE2A2A1} = 1$
96	Cyclin E:Cdk2(M)_E2F:hypo-pRb_E2F:hyper-pRb_Int -> E2F:hyper-pRb + Cyclin E:Cdk2(M)	Mass Action	$k_{upE2pRb} = 4.78271\ (min)^{-1}$
96_leuk	Cyclin E:Cdk2(M)_E2F:pseudo-hyper-pRb_E2F:hyper-pRb_Int -> E2F:hyper-pRb + Cyclin E:Cdk2(M)	Mass Action	$k_{upE2pRb_{leuk}} = pr_{upE2A2A1} * k_{upE2pRb}$<br>$pr_{upE2A2A1} = 0.428204395$

97	hypo-pRb + Cyclin A:Cdk2(M) -> Cyclin A:Cdk2(M)_hypo-pRb_hyper-pRb_Int	Mass Action	$k_{bA2pRb} = 6.25 \cdot e^{-5} \left(\left(\frac{molecules}{cell}\right) * min\right)^{-1}$
97_leuk	pseudo-hyper-pRb + Cyclin A:Cdk2(M) -> Cyclin A:Cdk2(M)_pseudo-hyper-pRb_hyper-pRb_Int	Mass Action	$k_{bA2pRb_{leuk}} = pr_{bE2A2A1} * k_{bA2pRb}$<br>$pr_{bE2A2A1} = 0.1301449788$
98	Cyclin A:Cdk2(M)_hypo-pRb_hyper-pRb_Int -> hypo-pRb + Cyclin A:Cdk2(M)	Mass Action	$k_{uA2pRb} = 0.1\ (min)^{-1}$
98_leuk	Cyclin A:Cdk2(M)_pseudo-hyper-pRb_hyper-pRb_Int -> pseudo-hyper-pRb + Cyclin A:Cdk2(M)	Mass Action	$k_{uA2pRb_{leuk}} = pr_{uE2A2A1} * k_{uA2pRb}$<br>$pr_{uE2A2A1} = 1$
99	Cyclin A:Cdk2(M)_hypo-pRb_hyper-pRb_Int -> hyper-pRb + Cyclin A:Cdk2(M)	Mass Action	$k_{upA2pRb} = 0.200091\ (min)^{-1}$
99_leuk	Cyclin A:Cdk2(M)_pseudo-hyper-pRb_hyper-pRb_Int -> hyper-pRb + Cyclin A:Cdk2(M)	Mass Action	$k_{upA2pRb_{leuk}} = pr_{upE2A2A1} * k_{upA2pRb}$<br>$pr_{upE2A2A1} = 0.428204395$
100	E2F:hypo-pRb + Cyclin A:Cdk2(M) -> Cyclin A:Cdk2(M)_E2F:hypo-pRb_E2F:hyper-pRb_Int	Mass Action	$k_{bA2pRb} = 6.25 \cdot e^{-5} \left(\left(\frac{molecules}{cell}\right) * min\right)^{-1}$
100_leuk	E2F:pseudo-hyper-pRb + Cyclin A:Cdk2(M) -> Cyclin A:Cdk2(M)_E2F:pseudo-hyper-pRb_E2F:hyper-pRb_Int	Mass Action	$k_{bA2pRb_{leuk}} = pr_{bE2A2A1} * k_{bA2pRb}$<br>$pr_{bE2A2A1} = 0.1301449788$
101	Cyclin A:Cdk2(M)_E2F:hypo-pRb_E2F:hyper-pRb_Int -> E2F:hypo-pRb + Cyclin A:Cdk2(M)	Mass Action	$k_{uA2pRb} = 0.1\ (min)^{-1}$
101_leuk	Cyclin A:Cdk2(M)_E2F:pseudo-hyper-pRb_E2F:hyper-pRb_Int -> E2F:pseudo-hyper-pRb + Cyclin A:Cdk2(M)	Mass Action	$k_{uA2pRb_{leuk}} = pr_{uE2A2A1} * k_{uA2pRb}$<br>$pr_{uE2A2A1} = 1$
102	Cyclin A:Cdk2(M)_E2F:hypo-pRb_E2F:hyper-pRb_Int -> E2F:hyper-pRb + Cyclin A:Cdk2(M)	Mass Action	$k_{upA2pRb} = 0.200091\ (min)^{-1}$
102_leuk	Cyclin A:Cdk2(M)_E2F:pseudo-hyper-pRb_E2F:hyper-pRb_Int -> E2F:hyper-pRb + Cyclin A:Cdk2(M)	Mass Action	$k_{upA2pRb_{leuk}} = pr_{upE2A2A1} * k_{upA2pRb}$<br>$pr_{upE2A2A1} = 0.428204395$
103	hypo-pRb + Cyclin A:Cdk1(M) -> Cyclin A:Cdk1(M)_hypo-pRb_hyper-pRb_Int	Mass Action	$k_{bA1pRb} = 6.73 \cdot e^{-5} \left(\left(\frac{molecules}{cell}\right) * min\right)^{-1}$
103_leuk	pseudo-hyper-pRb + Cyclin A:Cdk1(M) -> Cyclin A:Cdk1(M)_pseudo-hyper-pRb_hyper-pRb_Int	Mass Action	$k_{bA1pRb_{leuk}} = pr_{bE2A2A1} * k_{bA1pRb}$<br>$pr_{bE2A2A1} = 0.1301449788$
104	Cyclin A:Cdk1(M)_hypo-pRb_hyper-pRb_Int -> hypo-pRb + Cyclin A:Cdk1(M)	Mass Action	$k_{uA1pRb} = 0.1\ (min)^{-1}$
104_leuk	Cyclin A:Cdk1(M)_pseudo-hyper-pRb_hyper-pRb_Int -> pseudo-hyper-pRb + Cyclin A:Cdk1(M)	Mass Action	$k_{uA1pRb_{leuk}} = pr_{uE2A2A1} * k_{uA1pRb}$<br>$pr_{uE2A2A1} = 1$
105	Cyclin A:Cdk1(M)_hypo-pRb_hyper-pRb_Int -> hyper-pRb + Cyclin A:Cdk1(M)	Mass Action	$k_{upA1pRb} = 0.202132\ (min)^{-1}$
105_leuk	Cyclin A:Cdk1(M)_pseudo-hyper-pRb_hyper-pRb_Int -> hyper-pRb + Cyclin A:Cdk1(M)	Mass Action	$k_{upA1pRb_{leuk}} = pr_{upE2A2A1} * k_{upA1pRb}$<br>$pr_{upE2A2A1} = 0.428204395$
106	E2F:hypo-pRb + Cyclin A:Cdk1(M) -> Cyclin A:Cdk1(M)_E2F:hypo-pRb_E2F:hyper-pRb_Int	Mass Action	$k_{bA1pRb} = 6.73 \cdot e^{-5} \left(\left(\frac{molecules}{cell}\right) * min\right)^{-1}$
106_leuk	E2F:pseudo-hyper-pRb + Cyclin A:Cdk1(M) -> Cyclin A:Cdk1(M)_E2F:pseudo-hyper-pRb_E2F:hyper-pRb_Int	Mass Action	$k_{bA1pRb_{leuk}} = pr_{bE2A2A1} * k_{bA1pRb}$<br>$pr_{bE2A2A1} = 0.1301449788$
107	Cyclin A:Cdk1(M)_E2F:hypo-pRb_E2F:hyper-pRb_Int -> E2F:hypo-pRb + Cyclin A:Cdk1(M)	Mass Action	$k_{uA1pRb} = 0.1\ (min)^{-1}$
107_leuk	Cyclin A:Cdk1(M)_E2F:pseudo-hyper-pRb_E2F:hyper-pRb_Int -> E2F:pseudo-hyper-pRb + Cyclin A:Cdk1(M)	Mass Action	$k_{uA1pRb_{leuk}} = pr_{uE2A2A1} * k_{uA1pRb}$<br>$pr_{uE2A2A1} = 1$
108	Cyclin A:Cdk1(M)_E2F:hypo-pRb_E2F:hyper-pRb_Int -> E2F:hyper-pRb + Cyclin A:Cdk1(M)	Mass Action	$k_{upA1pRb} = 0.202132\ (min)^{-1}$
108_leuk	Cyclin A:Cdk1(M)_E2F:pseudo-hyper-pRb_E2F:hyper-pRb_Int -> E2F:hyper-pRb + Cyclin A:Cdk1(M)	Mass Action	$k_{upA1pRb_{leuk}} = pr_{upE2A2A1} * k_{upA1pRb}$<br>$pr_{upE2A2A1} = 0.428204395$
109	hyper-pRb -> pRb	Mass Action	$k_{tpRbDephos} = 0.023194\ (min)^{-1}$
109_leuk	pseudo-hyper-pRb -> pRb	Mass Action	$k_{tpRbDephos_{leuk}} = pr_{tpRbDephos} * k_{tpRbDephos}$<br>$pr_{tpRbDephos} = 1$
110	E2F:hyper-pRb -> E2F:pRb	Mass Action	$k_{tpRbDephos} = 0.023194\ (min)^{-1}$
110_leuk	E2F:pseudo-hyper-pRb -> E2F:pRb	Mass Action	$k_{tpRbDephos_{leuk}} = pr_{tpRbDephos} * k_{tpRbDephos}$<br>$pr_{tpRbDephos} = 1$
111	pRb + E2F -> E2F:pRb	Mass Action	$k_{bE2FpRb} = 9.66 \cdot e^{-6} \left(\left(\frac{molecules}{cell}\right) * min\right)^{-1}$
112	E2F:pRb -> pRb + E2F	Mass Action	$k_{uE2FpRb} = 0.1\ (min)^{-1}$
113	hypo-pRb + E2F -> E2F:hypo-pRb	Mass Action	$k_{bE2FpRb} = 9.66 \cdot e^{-6} \left(\left(\frac{molecules}{cell}\right) * min\right)^{-1}$
114	E2F:hypo-pRb -> hypo-pRb + E2F	Mass Action	$k_{uE2FpRb} = 0.1\ (min)^{-1}$
115	E2F:hyper-pRb -> hyper-pRb + E2F	Mass Action	$k_{uE2FpRb} = 0.1\ (min)^{-1}$
115_leuk	E2F:pseudo-hyper-pRb -> pseudo-hyper-pRb + E2F	Mass Action	$k_{uE2FpRb\_leuk} = pr_{uE2F} * k_{uE2FpRb}$<br>$pr_{uE2F} = 0.6127679966$
115_leuk_ reverse	pseudo-hyper-pRb + E2F -> E2F:pseudo-hyper-pRb	Mass Action	$k_{bE2FpRb\_leuk} = pr_{bE2F} * k_{bE2FpRb}$<br>$pr_{bE2F} = 1.88406857$
116	APCC + Emi1 -> APCC_Emi1	Mass Action	$k_{bEmi1APCC} = 0.0001\ (min)^{-1}$
117	APCC_Emi1 -> APCC + Emi1	Mass Action	$k_{uEmi1APCC} = 0.1\ (min)^{-1}$
118	CyclinA + APCC -> APCC_CyclinA_Int	Mass Action	$k_{bAPCCCyclinA} = 1.61 \cdot e^{-6} \left(\left(\frac{molecules}{cell}\right) * min\right)^{-1}$
119	APCC_CyclinA_Int -> CyclinA + APCC	Mass Action	$k_{uAPCCCyclinA} = 0.1\ (min)^{-1}$
120	APCC_CyclinA_Int -> APCC	Mass Action	$k_{udAPCCCyclinA} = 4.99955\ (min)^{-1}$
121	Cyclin A:Cdk2 + APCC -> APCC_Cdk 2_Cyclin A:Cdk2_Int	Mass Action	$k_{bAPCCCyclinA} = 1.61 \cdot e^{-6} \left(\left(\frac{molecules}{cell}\right) * min\right)^{-1}$
122	APCC_Cdk 2_Cyclin A:Cdk2_Int -> Cyclin A:Cdk2 + APCC	Mass Action	$k_{uAPCCCyclinA} = 0.1\ (min)^{-1}$

123	APCC_Cdk 2_Cyclin A:Cdk2_Int -> Cdk 2 + APCC	Mass Action	$k_{udAPCCCyclinA} = 4.99955\ (min)^{-1}$
124	p27:Cyclin A:Cdk2 + APCC -> APCC_p27:Cdk2_p27:Cyclin A:Cdk2_Int	Mass Action	$k_{bAPCCCyclinA} = 1.61 \cdot e^{-6} \left(\left(\frac{molecules}{cell}\right) * min\right)^{-1}$
125	APCC_p27:Cdk2_p27:Cyclin A:Cdk2_Int -> p27:Cyclin A:Cdk2 + APCC	Mass Action	$k_{uAPCCCyclinA} = 0.1\ (min)^{-1}$
126	APCC_p27:Cdk2_p27:Cyclin A:Cdk2_Int -> p27:Cdk2 + APCC	Mass Action	$k_{udAPCCCyclinA} = 4.99955\ (min)^{-1}$
127	Cyclin A:Cdk2(M) + APCC -> APCC_Cdk2(M)_Cyclin A:Cdk2(M)_Int; p27:Cyclin A:Cdk2	Mass Action	$k_{bAPCCCyclinA} = 1.61 \cdot e^{-6} \left(\left(\frac{molecules}{cell}\right) * min\right)^{-1}$
128	APCC_Cdk2(M)_Cyclin A:Cdk2(M)_Int -> Cyclin A:Cdk2(M) + APCC	Mass Action	$k_{uAPCCCyclinA} = 0.1\ (min)^{-1}$
129	APCC_Cdk2(M)_Cyclin A:Cdk2(M)_Int -> Cdk2(M) + APCC	Mass Action	$k_{udAPCCCyclinA} = 4.99955\ (min)^{-1}$
130	p27:Cyclin A:Cdk2(M) + APCC -> APCC_p27:Cdk2(M)_p27:Cyclin A:Cdk2(M)_Int	Mass Action	$k_{bAPCCCyclinA} = 1.61 \cdot e^{-6} \left(\left(\frac{molecules}{cell}\right) * min\right)^{-1}$
131	APCC_p27:Cdk2(M)_p27:Cyclin A:Cdk2(M)_Int -> p27:Cyclin A:Cdk2(M) + APCC	Mass Action	$k_{uAPCCCyclinA} = 0.1\ (min)^{-1}$
132	APCC_p27:Cdk2(M)_p27:Cyclin A:Cdk2(M)_Int -> p27:Cdk2(M) + APCC	Mass Action	$k_{udAPCCCyclinA} = 4.99955\ (min)^{-1}$
133	Cyclin A:Cdk1 + APCC -> APCC_Cdk1_Cyclin A:Cdk1_Int	Mass Action	$k_{bAPCCCyclinA} = 1.61 \cdot e^{-6} \left(\left(\frac{molecules}{cell}\right) * min\right)^{-1}$
134	APCC_Cdk1_Cyclin A:Cdk1_Int + APCC -> Cyclin A:Cdk1	Mass Action	$k_{uAPCCCyclinA} = 0.1\ (min)^{-1}$
135	APCC_Cdk1_Cyclin A:Cdk1_Int -> Cdk1 + APCC	Mass Action	$k_{udAPCCCyclinA} = 4.99955\ (min)^{-1}$
136	Cyclin A:Cdk1(M) + APCC -> APCC_Cdk1(M)_Cyclin A:Cdk1(M)_Int	Mass Action	$k_{bAPCCCyclinA} = 1.61 \cdot e^{-6} \left(\left(\frac{molecules}{cell}\right) * min\right)^{-1}$
137	APCC_Cdk1(M)_Cyclin A:Cdk1(M)_Int + APCC -> Cyclin A:Cdk1(M)	Mass Action	$k_{uAPCCCyclinA} = 0.1\ (min)^{-1}$
138	APCC_Cdk1(M)_Cyclin A:Cdk1(M)_Int -> Cdk1(M) + APCC	Mass Action	$k_{udAPCCCyclinA} = 4.99955\ (min)^{-1}$
**Additional reactions for Hypothesis Testing**			
139	-> Drug	Mass Action	r_{drug}
140	Drug + Cyclin D -> Drug:Cyclin D	Mass Action	$r_{drugBinding}$
			$ModifierTime = 675.9932642\ min$

(M): Activated species due to activating modifier switch activation

It is noted that varying precision number of decimal digits appears throughout Table 1. For the parameter values adopted from [1], the original precision has been kept. However, for the newly introduced parameters, the precision of the tool COPASI has been used and their estimated values are provided in Table 1 in their full precision in order to provide the reader with the possibility of reproducing the simulation outcome as closely to the presented results as possible.

1. Haberichter T, Mädge B, Christopher RA, Yoshioka N, Dhiman A, Miller R, Gendelman R, Aksenov SV, Khalil IG, Dowdy SF: **A systems biology dynamical model of mammalian G1 cell cycle progression**. *Mol Syst Biol* 2007, **3**:84.

Table S2 Initial levels of the model species

Species Name	Initial Levels $(molecules/cell)$
APCC	24582,9
APCC_Cdk1_Cyclin A:Cdk1_Int	104,388
APCC_Cdk1(M)_Cyclin A:Cdk1(M)_Int	0
APCC_Cdk 2_Cyclin A:Cdk2_Int	52,8191
APCC_Cdk2(M)_Cyclin A:Cdk2(M)_Int	0
APCC_p27:Cdk2_p27:Cyclin A:Cdk2_Int	90,5091
APCC_p27:Cdk2(M)_p27:Cyclin A:Cdk2(M)_Int	0
APCC_CyclinA_Int	8,79462
APCC_Emi1	5160,61
Cdk1	98550,6
Cyclin A:Cdk1	1345,01
Cdk1(M)	0
Cyclin A:Cdk1(M)	0
Cyclin A:Cdk1(M)_hypo-pRb_hyper-pRb_Int	0
Cyclin A:Cdk1(M)_E2F:hypo-pRb_E2F:hyper-pRb_Int	0
Cdk 2	33942
Cyclin E:Cdk2	2176,46
Cyclin A:Cdk2	680,557
Cdk2(M)	0
Cyclin E:Cdk2(M)	0
Cyclin E:Cdk2(M)_hypo-pRb_hyper-pRb_Int	0
Cyclin E:Cdk2(M)_E2F:hypo-pRb_E2F:hyper-pRb_Int	0
Cyclin A:Cdk2(M)	0
Cyclin A:Cdk2(M)_hypo-pRb_hyper-pRb_Int	0
Cyclin A:Cdk2(M)_E2F:hypo-pRb_E2F:hyper-pRb_Int	0
p27:Cdk2	58162
p27:Cyclin E:Cdk2	3729,51
p27:Cyclin A:Cdk2	1166,18
p27:Cdk2(M)	0
p27:Cyclin E:Cdk2(M)	0
p27:Cyclin A:Cdk2(M)	0
Cdk 4	46551,9
Cyclin D:Cdk 4	6547,64
Cyclin D:Cdk 4_pRb_hypo-pRb_Int	0
Cyclin D:Cdk 4_E2F:pRb_E2F:hypo-pRb_Int	0
p27:Cdk 4	41117,2
p27:Cyclin D:Cdk 4	5783,23
Cyclin A	113,316
Cyclin D	19264
Cyclin E	191,985
E2F	546,211
Emi1	248,046

p27	14150,9
pRb	58583,4
E2F:pRb	1416,59
hypo-pRb	0
E2F:hypo-pRb	0
hyper-pRb	0
E2F:hyper-pRb	0
pseudo-hyper-pRb	0
E2F:pseudo-hyper-pRb	0
Cyclin D:Cdk 4_ E2F:hypo-pRb_ E2F:pseudo-hyper-pRb_Int	0
Cyclin D:Cdk 4_hypo-pRb_pseudo-hyper-pRb_Int	0
Cyclin E:Cdk2(M)_pseudo-hyper-pRb_hyper-pRb_Int	0
Cyclin E:Cdk2(M)_E2F:pseudo-hyper-pRb_E2F:hyper-pRb_Int	0
Cyclin A:Cdk2(M)_pseudo-hyper-pRb_hyper-pRb_Int	0
Cyclin A:Cdk2(M)_E2F:pseudo-hyper-pRb_E2F:hyper-pRb_In	0
Cyclin A:Cdk1(M)_pseudo-hyper-pRb_hyper-pRb_Int	0
Cyclin A:Cdk1(M)_E2F:pseudo-hyper-pRb_E2F:hyper-pRb_Int	0
**Additional species for Hypothesis Testing**	
Drug	0
Drug_CyclinD	0

(M): Activated species due to activating modifier switch activation
