# Supplementary material for: Differentiation resistance through altered retinoblastoma protein function in acute lymphoblastic leukemia: in silico modeling of the deregulations in the G1/S restriction point pathway
Source: BMC Syst Biol. 2016 Mar 1;10:23. doi: 10.1186/s12918-016-0264-5 (PMC4774111; doi:10.1186/s12918-016-0264-5)
Supplement: Additional file 2: — Detailed description of the model calibration criteria derivation process. (PDF 458 kb) [file 12918_2016_264_MOESM2_ESM.pdf]

Additional file 2

Differentiation resistance through altered retinoblastoma protein function in Acute Lymphoblastic Leukemia: In silico modeling of the deregulations in the G1/S restriction point pathway

Eleftherios Ouzounoglou, Dimitra Dionysiou and Georgios S. Stamatakos

Detailed description of the model calibration criteria derivation process (referring to Figure 5 and Table 2 of the main manuscript)

Properties 1.a-c.

Although the bulk of pRb is found to be in this hyper-phosphorylated form in BCP-ALL primary cell extracts (property 1.a), its hypo-phosphorylated counterpart (Figure 5B) could be also detected (in contrast with stimulated CD34+ cells) in a significant percentage of cells (16% ± 13.2%) [1] (property 1.b). This may indicate that for the mean case this form of pRb is also present in at least detectable levels for a fraction of the G1-phase duration. Additionally, the existence of hypo-pRb is indicative of the presence of cells in early G1-phase. Based on that we speculate that leukemic cells also exhibit an early-G1-like phase during the first hours of their cell cycle. Unfortunately, due to the general lack in literature of time course and quantitative data for the exact levels of the different forms of pRb in BCP-ALL cells, the version of the protein that prevails during this alleged phase could not be elucidated. So, during the calibration of the model, specific constraints have been set in order for hypo-pRb to at least be higher than a detection threshold and to avoid complete disappearance for a pre-specified time span (hypo-pRb detection time span in Figure 5B), calculated as described later in this text. The detection threshold for hypo-pRb has been set using the simulation results of the reference model.

In more detail, in the experiments presented in [2], the hypo-pRb disappears 8 hours after the re-initiation of the cell cycle and so the levels returned from the reference model for this time point are adopted as the levels below which this form of the protein becomes un-detectable (approximately 3000 ($molecules/cell$)). Although this criterion has been formulated based on experiments done in a different cell type (colon carcinoma cell line), it is believed that it constitutes a realistic approximation for the detectable levels of hypo-pRb in any similar experiment.

Properties 2.f and 3a-g.

The transition to S-phase should be predicted to happen within a time range that the observed variance of the duration of cell cycle in BCP-ALL dictates. Although there is quantitative information about the duration of the whole cell cycle and of the S-phase in this type of cells, a specific measurement about the interval that the length of G1-phase spans was not found in the relevant literature. Therefore, since the model chosen to be modified covers only the execution of the G1 phase and the transition to S-phase, an estimation of the duration of G1-phase (or respectively of the time-point at which the G1/S-phase transition happens) should be made based on specific assumptions for the duration of M and G2 phases. This is feasible since there is concrete evidence that, in general, G1 is the most tunable phase of the cell cycle and majorly contributes to the tunability of its entire duration, in contrast with S, G2 and M phases which do not show significant temporal variances [3]. This is further supported by the fact that the duration of S-phase in precursor-B ALL cells is found to be identical to that of their normal counterparts [4].

In order to calculate the mean as well as a realistic range for the time point at which the transition into the S-phase can take place in BCP-ALL cells, we have taken into account the specific values for the mean duration of the cell cycle and the related phases in BCP-ALL cells, as dictated by the relevant literature (properties 3.a-3.d).

Therefore, taking the mean value of T_c we can estimate the relevant mean duration of the G1 phase (or equivalently the time point of transition into the S-phase), by subtracting the duration of the S, G2 and M phases from the whole cell cycle duration (property 3.e). Moreover, by adding or subtracting its standard deviation from the mean T_c value, the boundaries for a realistic range for G1 phase duration can be calculated in a similar way (property 3.e.).

Having calculated the mean G1-phase duration, the duration of the alleged early-G1-like phase (property 3.f.) could be estimated by taking into account the cell kinetics of BCP-ALL cells. More specifically, by knowing that approximately 94% of BCP-ALL cells are cycling ,2% are quiescent and 4% are apoptotic [1] (properties 2.a-c) and by subtracting the percentage of cells in S/G2/M phases (which is about 10%, property 2.d), the percentage of cells in G1 phase can be estimated to be approximately 84% (property 2.e), as is also stated in literature [4, 1, 5, 6]. Moreover, hypo-pRb is detected in about 16.3% of cells (property 1.b). So, by assuming that hypo-pRb is almost exclusively appointed in G1-phase, this percentage could be translated, by a simple analogy, to 13.7% of G1 cells being in an early-G1-like phase (property 2.f). Making the assumption that cells are distributed analogously to its duration in the G1 phase, for the mean case the cells should exist in the early-G1-like

phase for the 13.7% of G1 duration which can be rounded to approximately 800 minutes, again applying a simple analogy (property 3.f).

Properties 4a-4.d

The criterion for the passage into S-phase has been set to the increase of Cyclin A to levels similar to those reached in the reference model during the time needed for S-phase entrance (300 Cyclin A $molecules/cell$ at 10 hours, property 4.a). Again, these levels are believed to constitute a realistic approximation for Cyclin A levels required for the transition to S-phase, irrespectively of the cell type. Moreover, the G1/S transition is found undisturbed regarding the Cyclin A-related phenomena [5].Therefore, specific criteria had to be set in order to ensure that the trends of Cyclin A dynamics remain unchanged compared to those observed during the simulation of the reference model. More specifically, as can be seen in Figure 1, Cyclin A levels exhibit a period of stable or even decreasing levels until the activating modifier is switched on. So, for the newly proposed model, as depicted in Figure 5B, a specific criterion has been introduced in order for Cyclin A levels to remain below a specific threshold (150 $molecules/cell$) for this time interval, and in order to ensure that Cyclin A will not show any increasing trends for the same period of time, the rate of Cyclin A is set to remain negative or at least equal to zero (property 4.b). After the activating modifier is switched on, Cyclin A levels should start to elevate, but remain lower than 300 molecules since this level should be only reached when the time for G1/S transition has come (property 4.c). Finally, after passing the G1/S threshold, the levels of Cyclin A should continue to increase, since this type of Cyclin is majorly expressed during the

S and G2 phases of the cell cycle, which also stands true in the execution of the reference model. In order to formulate a related semi-quantitative criterion, the levels of Cyclin A returned by the reference model at the midpoint of S-phase duration (S-phase mid in Figure 5B, which for the case of the reference model is 3 hours) were sampled (470 $molecules/cell$, property 4.d) and the same levels are targeted to be reached by the newly proposed model, in 9 hours after passage to S-phase (the half of the S-phase duration in BCP-ALL).

References

1. Leibundgut K, Schmitz N, Tobler A, Lüthy AR, Hirt A: In childhood acute lymphoblastic leukemia the hypophosphorylated retinoblastoma protein, p110RB, is diminished, as compared with normal CD34+ peripheral blood progenitor cells. *Pediatr Res* 1999, 45(5 Pt 1):692–696.

2. Haberichter T, Mädge B, Christopher RA, Yoshioka N, Dhiman A, Miller R, Gendelman R, Aksenov SV, Khalil IG, Dowdy SF: A systems biology dynamical model of mammalian G1 cell cycle progression. *Mol Syst Biol* 2007, 3:84.

3. Holland-Frei Cancer Medicine [http://www.ncbi.nlm.nih.gov/books/NBK20777/]

4. Hirt A, Werren EM, Luethy AR, Gerdes J, Wagner HP: Cell cycle analysis in lymphoid neoplasia of childhood: differences among immunologic subtypes and similarities in the proliferation of normal and leukaemic precursor B cells. *Br J Haematol* 1992, 80:189–193.

5. Hirt A, Schmid AM, Julmy F, Schmitz NMR, Leibundgut K: Expression of cyclin A in childhood acute lymphoblastic leukemia cells reveals undisturbed G1-S phase transition and passage through the S phase. *Leuk Off J Leuk Soc Am Leuk Res Fund UK* 2009, 23:414–417.

6. Hirt A, Antic V, Wang E, Lüthy AR, Leibundgut K, Weid N von der, Tobler A, Wagner HP: Acute lymphoblastic leukaemia in childhood: cell proliferation without rest. *Br J Haematol* 1997, 96:366–368.
